# Quasi-Orthogonal Configuration of Propylene within a Scalable Metal–Organic Framework Enables Its Purification from Quinary Propane Dehydrogenation Byproducts

**DOI:** 10.1021/acscentsci.2c00554

**Published:** 2022-07-22

**Authors:** Peng Hu, Jialang Hu, Hao Liu, Hao Wang, Jie Zhou, Rajamani Krishna, Hongbing Ji

**Affiliations:** † Fine Chemical Industry Research Institute, School of Chemistry, Sun Yat-Sen University, Guangzhou 510275, People’s Republic of China; ‡ Van’t Hoff Institute for Molecular Sciences, University of Amsterdam, Park 904, 1098 XH Amsterdam, The Netherlands

## Abstract

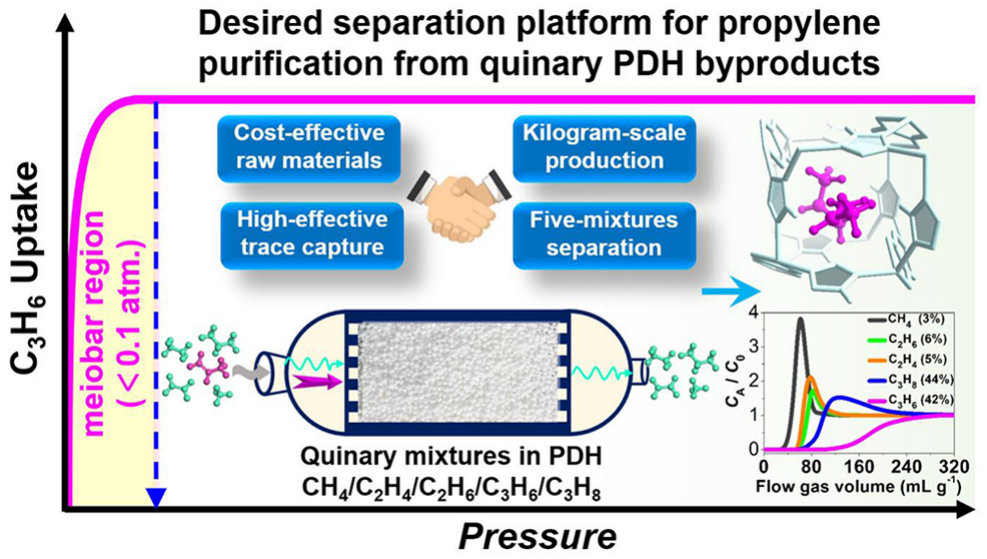

Propylene production via nonoxidative propane dehydrogenation (PDH) holds great promise in meeting growing global demand for propylene. Effective adsorptive purification of a low concentration of propylene from quinary PDH byproducts comprising methane (CH_4_), ethylene (C_2_H_4_), ethane (C_2_H_6_), propylene (C_3_H_6_), and propane (C_3_H_8_) has been an unsolved academic bottleneck. Herein, we now report an ultramicroporous zinc metal–organic framework (Zn-MOF, termed as **1**) underlying a rigid one-dimensional channel, enabling trace C_3_H_6_ capture and effective separation from quinary PDH byproducts. Adsorption isotherms of **1** suggest a record-high C_3_H_6_ uptake of 34.0/92.4 cm^3^ cm^–3^ (0.01/0.1 bar) at 298 K. In situ spectroscopies, crystallographic experiments, and modeling have jointly elucidated that the outstanding propylene uptakes at lower pressure are dominated by multiple binding interactions and swift diffusion behavior, yielding quasi-orthogonal configuration of propylene in adaptive channels. Breakthrough tests demonstrate that 30.8 L of propylene with a serviceable purity of 95.0–99.4% can be accomplished from equimolar C_3_H_6_/C_3_H_8_ mixtures for 1 kg of activated **1**. Such an excellent property is also validated by the breakthrough tests of quinary mixtures containing CH_4_/C_2_H_4_/C_2_H_6_/C_3_H_6_/C_3_H_8_ (3/5/6/42/44, v/v/v/v/v). Particularly, structurally stable **1** can be easily synthesized on the kilogram scale using cheap materials (only $167 for per kilogram of **1**), which is important in industrial applications.

## Abstract 2

We report a scalable ultramicroporous Zn-MOF with rigid one-dimensional channels using cheap raw materials, enabling trace propylene capture and its separation from quinary PDH byproducts.

## 1 Introduction

Propylene (C_3_H_6_), as one of the most important chemical products, is widely used in the production of various chemicals, including polymers (e.g., polypropylene) and oxygenates (e.g., acetone and propylene oxide) etc.,^1,2^ and is expected to grow above 130 million metric tons by 2023.^3^ In the petrochemical industry, nonoxidative propane dehydrogenation (PDH) is becoming popular and is regarded as a promising way to meet the ever-increasing demand for propylene across the globe.^4^ The resultant byproducts in PDH reactions including methane (CH_4_), ethane (C_2_H_6_), ethylene (C_2_H_4_), and propane (C_3_H_8_) impurities doubtlessly reduce the purity and productivity of C_3_H_6_. Effective C_3_H_6_ separation from binary C_3_H_6_/C_3_H_8_ mixtures (only 0.04 nm in kinetic diameter, Table S1) and even quinary mixtures in PDH byproducts (typically consisting of ca. 1–3% CH_4_, 0.5–6% C_2_H_6_, 0.2–5% C_2_H_4_, 40–45% C_3_H_8_, and 42–50% C_3_H_6_) is a prerequisite for improving the quality of propylene. The separation technologies currently used are based on energy- and capital-intensive distillation operation, certainly contributing to larger energy loss and economic costs. Adsorbent-based separation strategies could theoretically alleviate the above-mentioned energy consumption without a phase change, namely, distinguishing the gas molecules only through molecule size, shape, polarity, and other characteristics.^5^

**Scheme 1:**
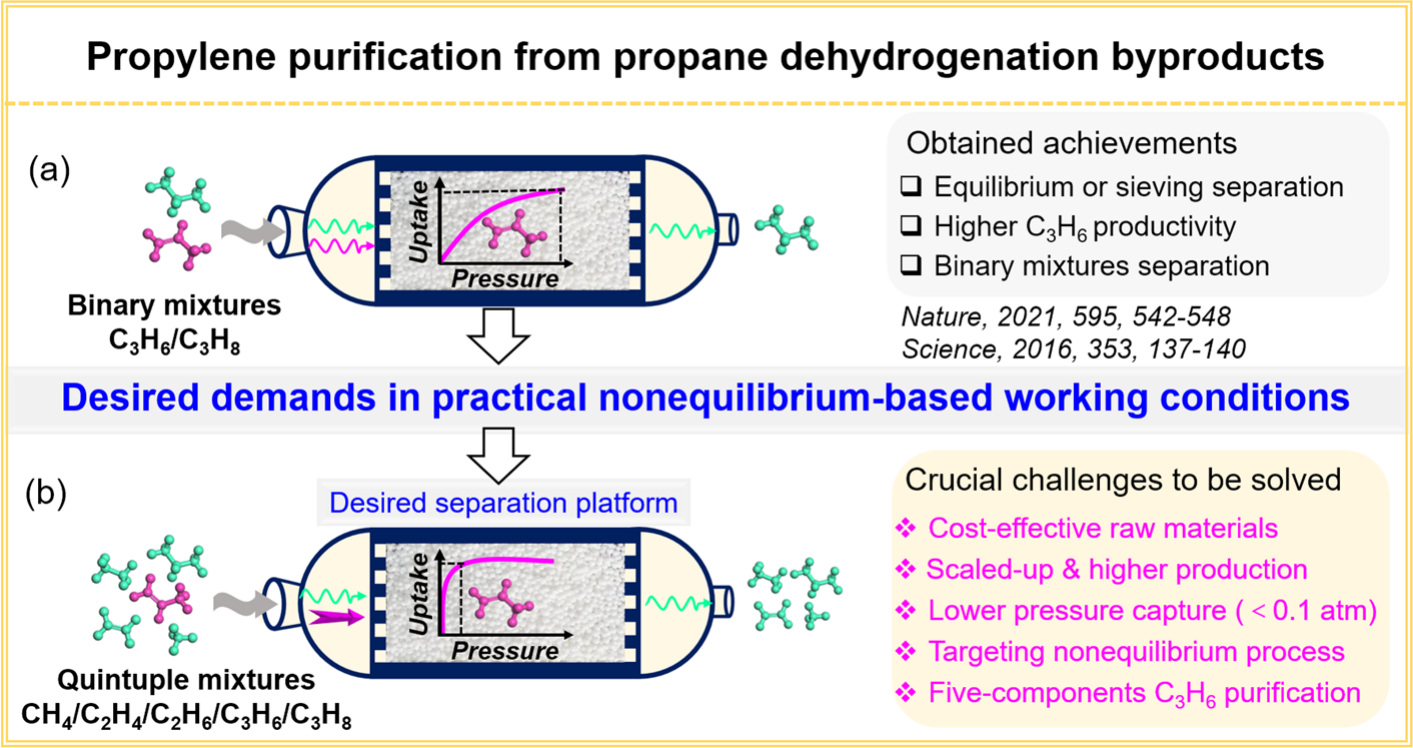
Schematic Illustration of Propylene Purification from Propane Dehydrogenation (PDH) Byproducts, Including (a) Obtained Academic Achievements for Binary C_3_H_6_/C_3_H_8_ Mixtures and (b) Crucial but Neglected Challenges for C_3_H_6_ Purification from PDH Byproducts Containing Quinary CH_4_/C_2_H_4_/C_2_H_6_/C_3_H_6_/C_3_H_8_ Mixtures

Metal–organic frameworks (MOFs), as well-known porous adsorbents, have been extensively explored for gas separation due to their adjustable pore chemistry and structural diversity etc.^6−9^ In terms of separation mechanisms, it can be roughly divided into thermodynamic separation and nonequilibrium separation. Compared with unilateral thermodynamic equilibrium dominated by binding affinities, the strategies that rely on nonequilibrium separation (including sieving separation and kinetic-driven separation) can be more energy-efficient and realistic given the fact that industrial pressure swing adsorption (PSA), vacuum swing adsorption (VSA), and temperature swing adsorption (TSA) processes are actually operating under nonequilibrium operating conditions. For example, Chen et al.^10^ covered the Co-gallate for sieving separation of C_3_H_6_/C_3_H_8_, which suggested a notable adsorption capacity of 66.6 cm^3^ cm^–3^ at 1 bar and 298 K. Breakthrough tests revealed the high purity of propylene (97.7%) with a high dynamic separation productivity of 36.4 cm^3^ cm^–3^ under ambient conditions. Another praiseworthy sieving stage, JNU-3a, designed by Li et al.,^11^ featured one-dimensional channels with embedded molecular pockets and realized the sieving separation of binary C_3_H_6_/C_3_H_8_, yielding high-purity C_3_H_6_ (≥99.5%) and a C_3_H_6_ productivity of 53.5 L kg^–1^. Unfortunately, strong sieving restriction in the pore channels might cause some unavoidable issues associated with the diffusion behavior and regeneration process. From the perspective of structural flexibility, most linkers are flexible in nature; accurately controlling pore size within a critical range to fully sieve C_3_H_6_ from C_3_H_6_/C_3_H_8_ mixtures is still in its infancy. Conversely, the kinetic effects, a significative diffusion-driven mechanism in the nonequilibrium process, could be dexterously designed to effectively accomplish the nonequilibrium separation. Li et al.^12^ prepared a MOF (termed as ELM-12), showing an enhanced C_3_H_6_ uptake of 62.0 mg g^–1^ at 298 K and 1 bar and higher kinetic C_3_H_6_/C_3_H_8_ selectivity (204 at 298 K and 971 at 308 K). Breakthrough tests also confirmed the separation performance for binary mixtures, yielding a C_3_H_6_ productivity of 457 mmol per liter. Also, the Li and Eddaoudi groups developed several MOFs having good kinetic selectivity that could be used for binary C_3_H_6_/C_3_H_8_ separation.^13,14^ Of particular note is that, albeit conspicuous achievements have been achieved for kinetic separation of C_3_H_6_/C_3_H_8_, regrettably, these studies have only focused on binary mixtures. Synergistic kinetic-driven separation of C_3_H_6_ from quinary mixtures, especially from PDH byproducts containing CH_4_, C_2_H_6_, C_2_H_4_, and C_3_H_8_ impurities, has not been realized yet. Another crucial but easily overlooked fact is that the partial pressure of propylene is usually low (<300 mbar) in quinary mixtures,^15^ which undoubtedly poses a serious challenge for trace C_3_H_6_ capture and C_3_H_6_ purification at a low partial pressure (Scheme 1).

Given the concerns mentioned above, we now report the first paradigm of using an ultramicroporous zinc metal–organic framework (Zn-MOF, termed as **1**) with rigid one-dimensional channels and decent pore chemistry for trace C_3_H_6_ capture and its purification from quinary PDH byproducts containing CH_4_, C_2_H_6_, C_2_H_4_, C_3_H_6_, and C_3_H_8_ mixtures. Static adsorption isotherms suggest that **1** possesses the record-high C_3_H_6_ uptake of 92.4 cm^3^ cm^–3^ at 298 K and 0.1 atm, indicating a great potential for C_3_H_6_ capture at lower partial pressure. Further, comprehensive analysis including in situ spectroscopies, crystallographic experiments, and modeling analysis have cooperatively demonstrated that the decent pore microenvironment and multiple task-specific groups enabled synergetic equilibrium effects and “sweet spots” of kinetics for trapping C_3_H_6_, evidently boosting C_3_H_6_ separation. In particular, two C_3_H_6_ molecules adsorbed in one unit cell exhibit an unusual quasi-orthorhombic configuration, which favors the intramolecular interaction and multiple binding models with pore pockets. Breakthrough tests demonstrate that **1** is capable of separating high-purity C_3_H_6_ (95.0–99.4%) from an equimolar C_3_H_6_/C_3_H_8_ mixture under ambient conditions, giving a maximum C_3_H_6_ productivity of 30.8 L for 1 kg of activated **1** under ambient conditions. The excellent separation property of C_3_H_6_ on **1** is also validated by the experimental and simulated breakthrough tests of quinary PDH byproducts containing CH_4_/C_2_H_4_/C_2_H_6_/C_3_H_6_/C_3_H_8_ (3/5/6/42/44, v/v/v/v/v) mixtures, suggesting that **1** could inherit the preferable separation performance for trapping C_3_H_6_ from PDH byproducts. Notably, **1** possesses good structural stability and can be easily synthesized on the kilogram scale using cheap raw materials (only $167 for per kilogram of **1**), awarding **1** the potential benchmark stage to purify C_3_H_6_ from multiple components.

## 2 Experimental Section

### 2.1 Materials

All reagents and solvents were purchased commercially and used without further processing. Zinc oxalate and 1,2,4-triazole were purchased from Macklin, Shanghai, China. Ethanol (C_2_H_5_OH, 99.5%) and methanol (CH_3_OH, 99.5%) were purchased from Aladdin Industrial Co., Ltd., Shanghai, China.

### 2.2 Scalable Synthesis of Robust Zn-MOF (**1**)

**1** was prepared according to the following method with some modifications.^16^ In detail, zinc oxalate and 1,2,4-triazole were mixed at the molar ratio of 1:2.3, then added into aqueous solution containing methanol/ethanol and ultrasonic stirring. Subsequently, the solutions were transferred to a Teflon autoclave and heated at 453 K for 72 h. The yielded products were then washed with methanol and ethanol and then heated in a vacuum oven at 373 K for 12 h to afford desolvated **1**.

### 2.3 Dynamic Column Breakthrough Experiments

Dynamic breakthrough experiments were tested in a homemade breakthrough setup and monitored on a gas chromatograph (GC). Prior to the breakthrough experiments, 0.3 g of activated **1** adsorbent was filled into the customized adsorption column (7.0 mm I.D. and 250 mm in length); glass wool was used to plug the two ends of the column. Then, the column was in situ heated at a temperature of 373 K for 12 h with a helium flow (5 sccm) to remove the adsorbed gas impurities. After the system was stabilized, the device was cooled to 298 K, and the gas mixtures of C_3_H_6_/C_3_H_8_/He (30/30/40, v/v/v) were introduced into the pipeline. The gas mixtures were passed through the column at a flow rate of 2 sccm and detected through GC. In the regeneration procedure, the adsorbent was in situ heated at 373 K for 12 h through using sweeping He gas at a rate of 5 sccm. For quinary CH_4_/C_2_H_4_/C_2_H_6_/C_3_H_6_/C_3_H_8_ (3/5/6/42/44, v/v/v/v/v), 0.01 kg of **1** was filled into the customized adsorption column (21.0 mm I.D. and 250 mm in length; note that the adsorbents were extruded, ground, and sieved into 40–60 mesh particles to minimize the impacts of diffusion and pressure drop). Other procedures were kept the same.

The captured capacity of gas on **1** could be estimated using eq 1:
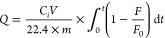
1where *Q* is the equilibrium adsorption capacity of gas *i* (mmol g^–1^), *C*_*i*_ is the feed gas concentration, *V* is the volumetric feed flow rate (sccm), *t* is the adsorption time (min), *F*_0_ and *F* are the inlet and outlet gas molar flow rates, respectively, and *m* is the mass of the adsorbent (g).

## 3 Results and Discussion

### 3.1 Structural Analysis of Robust **1**

**Figure 1:**
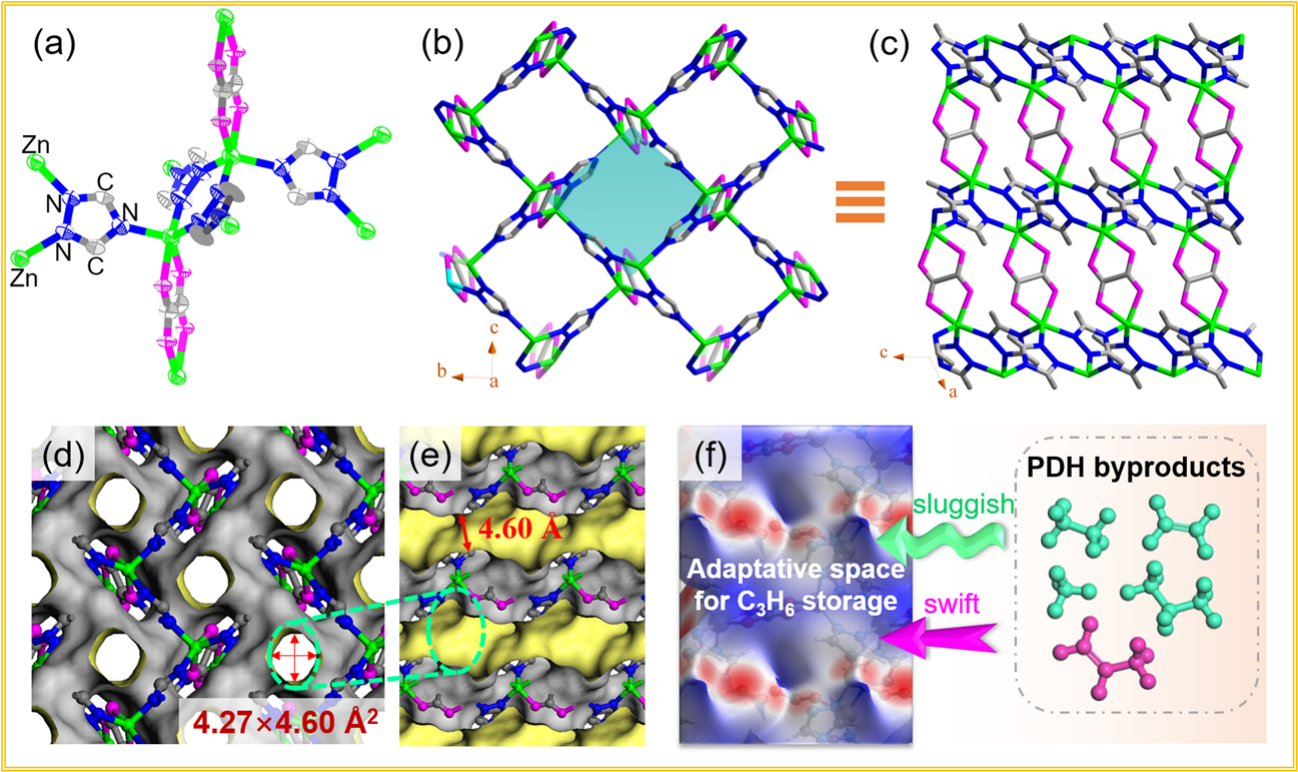
(a) ORTEP plot of the single-crystal X-ray structure of the **1** model with probability ellipsoids drawn at 80%. (b) 3D crystal structure of **1** along the *a* axis. (c) View orthogonal to b showing the pillaring of the zinc triazolate layers by oxalate anions (the different nets are highlighted in bule, pink, bright blue, and gray-40% for clarity (code mode: C in ligands, gray-40%; Zn, bright blue; O, pink). (d) Accessible Connolly surface representation of one-dimensional pore geometry along the *a* axis, with an aperture size of 4.27 × 4.60 Å^2^ (excluding van der Waals radii). (e) Diagram of diffusion channels for various guests and (f) the Hirshfeld surface (de) displaying the favorable electrostatic potential for C_3_H_6_ capture from PDH byproducts containing CH_4_, C_2_H_6_, C_2_H_4_, C_3_H_6_, and C_3_H_8_ mixtures, of which C_3_H_6_ was drawn in pink and other guests were drawn in green for clarity.

In the case of single unit structure, the Zn center was five-coordinated with a distorted trigonal bipyramidal geometry (Figure 1a). In detail, the nitrogen atoms located in the 1,2-positions of the triazolate coordinated with Zn dimers and further connected to the next dimer via the nitrogen atom in the 4-position of the triazolate, as a result, forming into two-dimensional (2D) lattice planes. Interestingly, the layers of 1,2,4-triazolate-bridged zinc(II) were further pillared by oxalate species to construct the three-dimensional (3D) lattice and 3D pore geometry (Figure 1b,c; a detailed list of atomic positions for the Zn-MOF model are available in Table S2). The crystalline phase and purity of as-prepared bulk **1** was verified by combing the comparisons of theoretical and experimental PXRD diffractions. As clearly shown in Figure S1, all of the experimentally measured characteristic peaks agreed well with the simulated data and crystallized in the *P*21/*c* space group, indicating the isostructural topological structure of **1**. Further, the cell volumes derived from refinement analysis between experimental **1** and the theoretical model gave a Δ*V*/*V* of 0.005% (Table S3), being much lower than that of reported rigid MOF-5 (0.8%).^17^ The quais-unchanged cell volume shrinkage revealed the excellent skeleton rigidity of **1**, although indirectly. In order to confirm the structural rigidity under variable-temperature conditions, we conducted PXRD tests at 298 and 373 K to investigate the evolution of the unit cell. As shown in Figure S2a,b, it is suggested that there are no apparent shifts in the positions of all peaks for **1**, revealing a higher framework lattice rigidity and structure stability without phase changes observed. In addition, the cell volumes of **1** at 298 (Figure S2a) and 373 K (Figure S2b) afforded a Δ*V*/*V* of 0.02% (Table S3). Intuitively, the cell structures of **1** underwent quasi-unchanged conformation deformations (Figure S2c,d) compared with that of theoretical topology, suggesting a credible structure stability. The excellent structure stability was also demonstrated by TGA analysis (Figure S3), yielding a higher decomposition temperature exceeding 600 K.

Intuitional visualization of pore conformation, the simulated Connolly pore surface exhibited a zigzag shaped one-dimensional pore geometry along the crystallographic *a* axis, with a cross-sectional size of ca. 4.27 × 4.60 Å^2^ (Figure 1d,e; excluding the distances of the van der Waals radii), which would be favorable for distinguishing the gas diffusion behaviors. Especially for C_3_H_6_ and C_3_H_8_ guests with highly similar molecular attributes, the decent pore window seemed to be unfulfillable for both C_3_H_8_ (with the kinetic diameter of 5.1 Å) and C_3_H_6_ (4.7 Å) to diffusion into the pore channels from the kinetic diameter point of view. Nevertheless, the suitable pore space and the van der Waals molecular dimension differentiation could be expected to realize the separation of C_3_H_8_ (4.20 × 4.80 × 6.80 Å^3^) and C_3_H_6_ (4.16 × 4.65 × 6.45 Å^3^; Table S1). Thus, the propylene molecule with a minimum cross-section size of 4.16 × 4.65 Å^2^ or 19.34 Å^2^ could theoretically diffuse into the channel of **1** with a contented cross-section size (4.27 × 4.60 Å^2^ or 19.64 Å^2^), while propane with a minimum cross-section size of 4.20 × 4.80 Å^2^ or 20.16 Å^2^ would be excluded from the pore channels due to the limited cross-section size of **1**. Note that this slight shape sieving may not achieve an ideal sieving effect, but it is important to influence the diffusion behavior of molecules. A deep insight into the Hirshfeld surface (Figure 1f) is that it can be observed that a highly attractive negative electrostatic potential mapped with −0.05 au (red) was clearly distributed in the pore channel, suggesting the enriched pore polarity, which favored the binding interaction with the molecule that had a larger dipole moment.^18,19^ In brief, due to decent molecule dimensions and the large dipole moment of C_3_H_6_, it is expected to form strong interactions with the polar pore surface and will effortlessly diffuse into the adaptive pore channel.

### 3.2 Isothermal Adsorption and Selectivity Analysis

**Figure 2:**
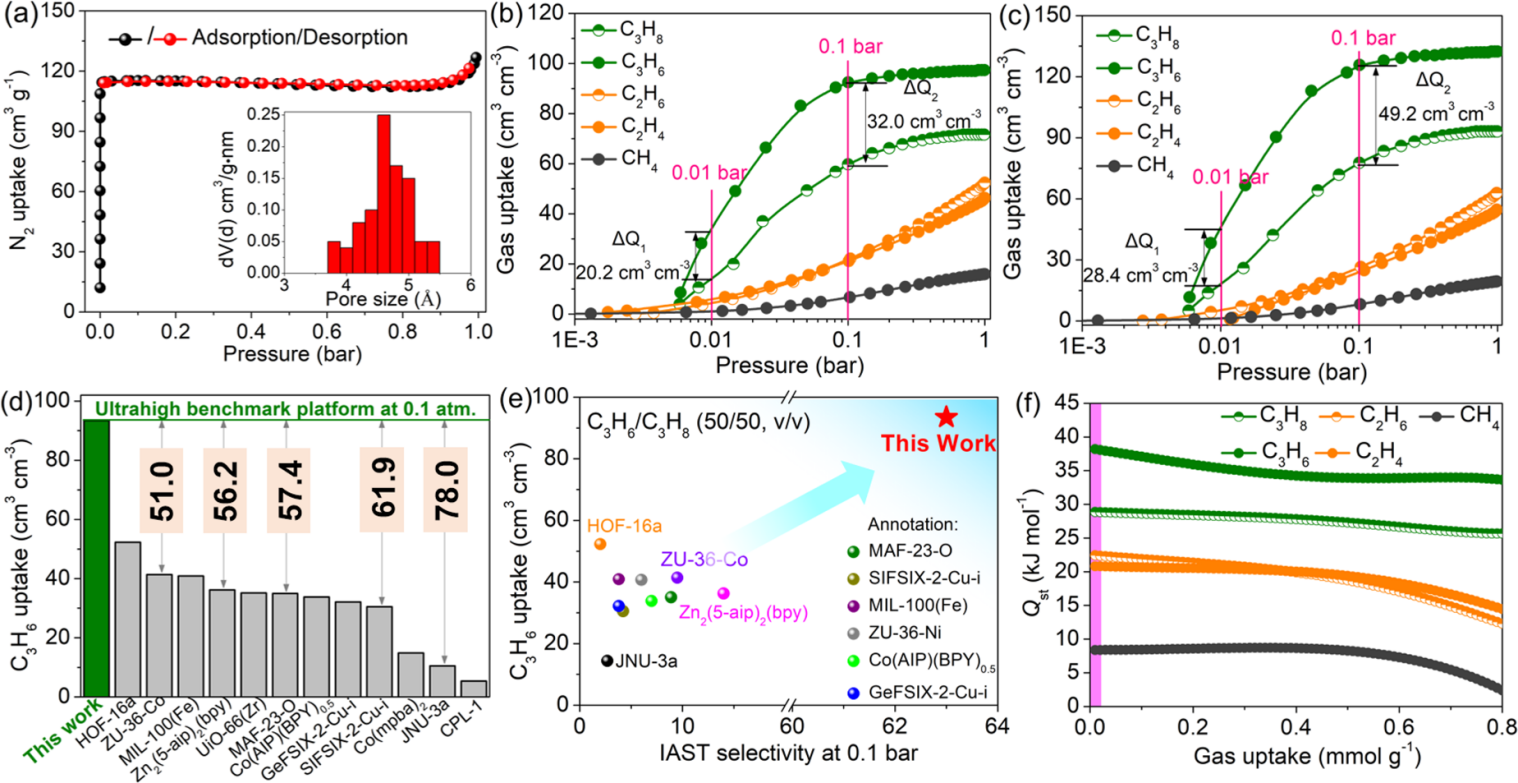
(a) N_2_ adsorption isotherms at 77 K and 1 bar. (b,c) Single-gas adsorption isotherms of various molecules at (b) 298 K and (c) 273 K, respectively, with a pressure of 1 bar. (d) Comparisons of volumetric uptake of C_3_H_6_ on **1** and well-known adsorbents at 298 K and 0.1 bar. (e) Comparisons of IAST selectivity of C_3_H_6_/C_3_H_8_ (50/50, v/v) versus C_3_H_6_ uptake on **1** and benchmark materials at 298 K and 0.1 bar. (f) Coverage-dependent adsorption enthalpy profiles of various guest molecules on **1** obtained by the virial fitting method.

The eternal pore attributes of **1** were determined at 77 K through using N_2_ as the probe molecule. As clearly seen in Figure 2a, **1** exhibited a representative I-type profile, yielding a N_2_ capacity of 127.4 cm^3^ g^–1^ at 1 atm. The Brunauer–Emmett–Teller (BET) surface area and pore volume were evaluated to be 526.1 m^2^ g^–1^ and 0.35 cm^3^ g^–1^ by adopting the ASAP 2020 physisorption analyzer, being nearly identical to the theoretical values of 510.8 m^2^ g^–1^ and 0.29 cm^3^ g^–1^ (calculated from the optimized crystal structure). The pore size distribution (PSD) according to the Horvath–Kawazoe model revealed the ultramicropore with a peak centered at ca. 4.62 Å (inset in Figure 2a). The permanent ultraporosity and the decent pore dimensions motivated us to explore the potential adsorption performance of propane dehydrogenation byproducts including CH_4_, C_2_H_4_, C_2_H_6_, C_3_H_6_, and C_3_H_8_ on activated **1**. We collected the single-component adsorption isotherms of various guests on **1** at 298 K up to 1 atm. Here, volumetric uptake is adopted to evaluate the adsorption performance of the adsorbents in industry as that would determine the footprint of the gas separation units.^20^ As clearly depicted in Figure 2b, **1** exhibited a distinguished C_3_H_6_ adsorption steepness at lower concentrations of 0.01–0.1 bar, giving the ultrahigh volumetric C_3_H_6_ uptakes of 34.0/92.4 cm^3^ cm^–3^ (0.01/0.1 bar) at 298 K, as revealed by the steepness of the C_3_H_6_ adsorption isotherms. Conversely, it indicated eclipsed adsorption capacity for other guest molecules under identical conditions (Figure 2b). Further increasing pressure to 1 bar, **1** suggested quasi-saturated capacity of 97.4 cm^3^ cm^–3^, corresponding to 9.4 wt % or 1.94 C_3_H_6_ molecule per cell unit. In addition, the differences of statical adsorption capacity (denoted as ΔQ) for C_3_H_6_ and C_3_H_8_ at a lower pressure of 0.01/0.1 bar afforded Δ*Q*_1_ and Δ*Q*_2_ values of 20.2 and 32.0 cm^3^ cm^–3^ at 298 K (Figure 2b), confirming the preference for trapping trace C_3_H_6_ under lower partial pressure. Such differences could be visually observed at 273 K (Figure 2c). Apparently, **1** revealed the enhanced C_3_H_6_ uptake, with values of 45.3/125.4/133.4 cm^3^ cm^–3^ (0.01/0.1/1 bar), and the Δ*Q* values between C_3_H_6_ and C_3_H_8_ were up to 28.4/49.2/40.0 cm^3^ cm^–3^ for 0.01/0.1/1 bar, respectively. In addition, according to the saturated C_3_H_6_ capacity, the density of gaseous C_3_H_6_ in **1** was determined to be 281.3 g L^–1^ at 298 K and 0.1 bar. To our knowledge, the storage density of confined C_3_H_6_ in the channel far surpassed that of other benchmark materials including Zn_2_(5-aip)_2_(bpy) (135.9 g L^–1^),^20^ SIFSIX-2-Cu-i (135.5 g L^–1^),^22^ and CPL-1 (43.7 g L^–1^)^23^ etc. Such a higher storage density of C_3_H_6_ at 0.1 atm was more than 164-fold higher than that of gaseous C_3_H_6_ (1.707 g L^–1^) under similar conditions, suggesting that cooperative stacking models or intramolecular binding affinities may be responsible for C_3_H_6_ capture under lower pressure. Such an unusual adsorption configuration for trace C_3_H_6_ capture on **1** was mainly attributed to the larger polarizability/dipole moment of C_3_H_6_ (Table S1), which exerted a crucial effect on the purification of propylene at lower concentrations, especially for propane cracking-gas mixtures containing multiple components.

In order to further highlight the advantages of **1** at low pressure, detailed comparisons with other advanced materials were integrated at 298 K and 0.1 bar. Results evidenced that **1** remained the record-high volumetric capacity of C_3_H_6_ (92.4 cm^3^ cm^–3^), surpassing that of most state-of-the-art competitors, including the newly reported MOF platforms ZU-36-Co (41.4 cm^3^ cm^–3^),^24^ Zn_2_(5-aip)_2_(bpy) (36.2 cm^3^ cm^–3^),^21^ and JNU-3a (14.4 cm^3^ cm^–3^),^25^ etc. (Figure 2d). To quantitatively evaluate the separation potential of **1** for binary C_3_H_6_/C_3_H_8_ mixtures, the ideal adsorbed solution theory (IAST) selectivity was evaluated using the double site Langmuir–Freundlich (DSLF) model (Figure S4a,b and Table S5).^26,27^ Obviously, **1** indicated a remarkable C_3_H_6_/C_3_H_8_ IAST selectivity, with a value of 63 at 0.1 bar (Table S4), far ascendant to many C_3_H_6_-selective prototypes covered, including MAF-23-O (8.9),^28^ Zn_2_(5-aip)_2_(bpy) (14.2),^21^ and MIL-100(Fe) (3.8).^29^ In addition, the comprehensive comparisons between C_3_H_6_ uptakes and the selectivity of C_3_H_6_/C_3_H_8_ at 0.1 bar suggested that **1** ranked in the “ceiling” level (Figure 2e), uncovering that **1** can effectively overcome the obstacles in balancing insurmountable “trade-off” effects. The unwonted breakthrough in trapping trace C_3_H_6_ is of the utmost importance, especially from multiple components with lower partial pressures of propylene. Note that IAST methods are often subject to uncertainties and limited requirements, and large errors can arise from narrow pores (nonideal gas solution), framework flexibility, a large binding difference, etc. So, the IAST selectivites were calculated here just for qualitative comparison.^30^ The coverage-dependent isosteric adsorption heats (*Q*_st_) were calculated using the virial method (Figure 2f and Table S6) to explore the interaction energies between various molecules and the host framework. Apparently, the *Q*_st_ of **1** at zero coverage followed the hierarchy of C_3_H_6_ (38.3 kJ mol^–1^) > C_3_H_8_ (28.9 kJ mol^–1^) > C_2_H_6_ (22.4 kJ mol^–1^) > C_2_H_4_ (20.7 kJ mol^–1^) > CH_4_ (8.2 kJ mol^–1^; Figure 2f), conferring **1** with the obvious potential stage to capture C_3_H_6_ from propane dehydrogenation byproducts. To be noted, the *Q*_st_ of C_3_H_6_ at zero coverage was much lower than that of known Zn_2_(5-aip)_2_(bpy) (46 kJ mol^–1^)^21^ and MAF-23-O (54 kJ mol^–1^),^28^ being comparable to SIFSIX-2-Cu-I (35.8 kJ mol^–1^)^22^ and ZU-36-Co (38.0 kJ mol^–1^).^31^ The desorption activation energy of C_3_H_6_ obtained from the TPD profiles was calculated to be 41.5 kJ mol^–1^ (Figure S5a,b), also evidently confirming the strong binding affinity between C_3_H_6_ and the framework. Such a low adsorption enthalpy not only awarded the **1** platform a lower regeneration energy to yield higher C_3_H_6_ productivity but also avoided C_3_H_6_ oligomerization/polymerization that may damage the binding sites.

### 3.3 Adsorption Conformation and Binding Mechanism

**Figure 3:**
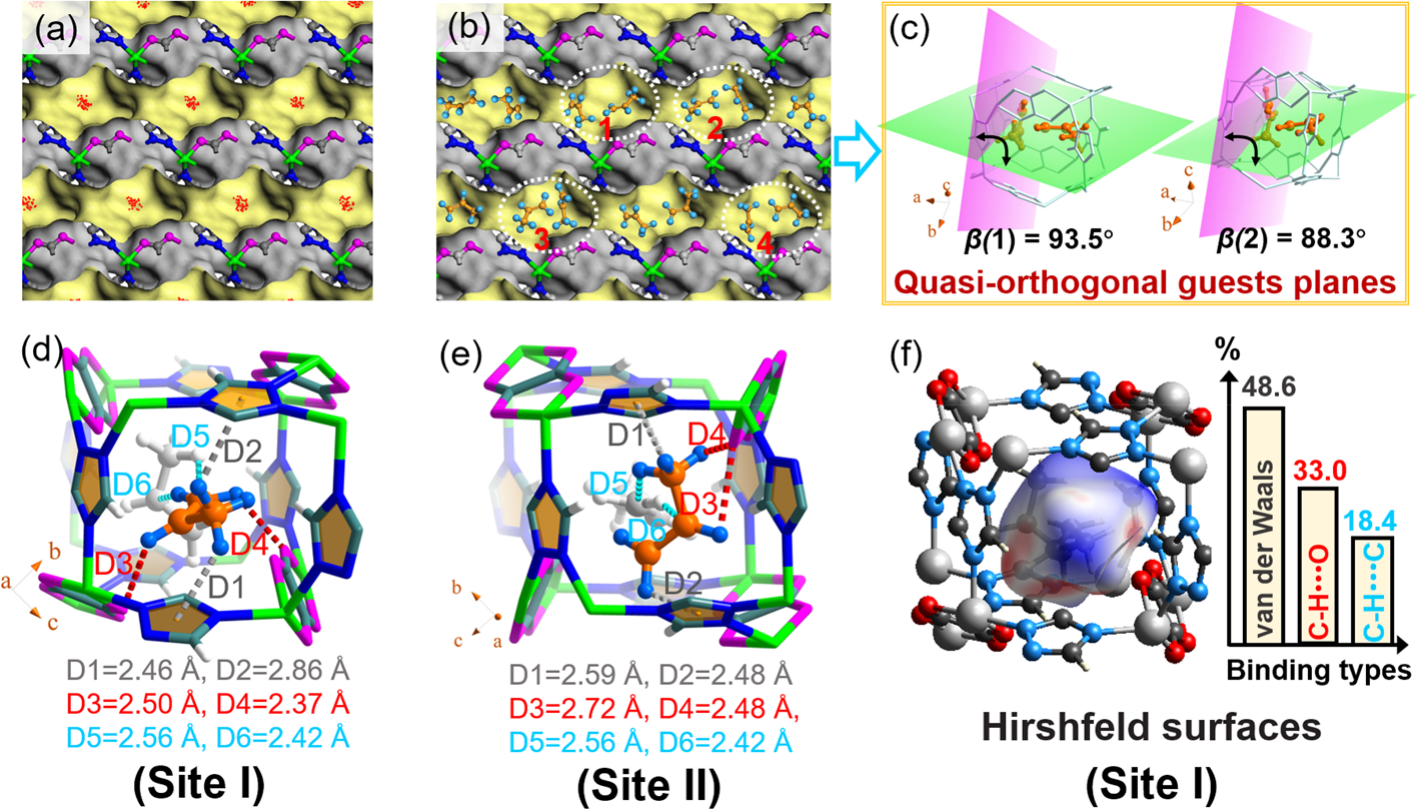
(a) Density distribution of C_3_H_6_ molecules within **1** topology obtained from GCMC simulations. (b) Side views of the packing diagram of the C_3_H_6_ adsorbed in the framework of **1**. (c) Visualized planes of guest molecule yielded from b (**1** and **2**, marked with red color) and created by three C atoms on a molecule (the atoms in guest molecules are highlighted with orange and other atoms in MOF structure are highlighted with light turquoise for clarity). (d) DFT calculated adsorption conformation of C_3_H_6_-loaded **1** in site I and (e) DFT calculated adsorption conformation of C_3_H_6_-loaded **1** in site II (note that the binding types are colored in gray, red, and turquoise, corresponding to van der Waals interaction, hydrogen-bonding, and intramolecular forces; color modes: H in ligands, white; H in guest molecule, light blue; Zn, bright green; O, pink; N, blue; C, sea green). (f) Hirshfeld surface (de) displaying host–guest interactions of C_3_H_6_-loaded **1** in site I.

To deeply elucidate the binding sites, we adopted Grand Canonical Monte Carlo (GCMC) simulations to investigate the binding models and adsorption mechanism between guest molecules and **1**. As obviously observed, the simulated adsorption isotherms on **1** agreed well with the experimental results on the overall trend at 298 K, although some points at ultralow pressure and higher pressure were not particularly intimate (Figure S6). The inconsistent paces may derive from the strong binding affinity between guest molecules and the framework, which were intractable to construct the topology models using simple force fields. Additionally, the visualized density distribution contours of C_3_H_6_-loaded **1** (Figure 3a) suggested that the adsorbed C_3_H_6_ was concentrated and arranged in the pore space one by one in a straight line along the zigzag shaped open channels. The optimized C_3_H_6_ configurations obtained from GCMC-simulated saturated capacity also indicated 2-fold disordering over two binding conformations with partial occupancy, which oriented linearly with its C=C axis along the channels and tilted with its minimum cross-section along the pore wall (Figure 3b). The saturated adsorption orientation in the pore pocket would minimize any possible steric hindrance and electrostatic repulsion from the polar framework. Further, the spatial stacking conformation of two C_3_H_6_ molecules located at labels **1** and **2** (Figure 3b, highlighted with red color) were amplified and shown in geometrical-plane perspectives (Figure 3c). Note that in order to intuitively mirror the stacking models of the two molecules, the C=C–C bond in a single molecule was selected as the reference point to construct the conformational plane. Interestingly, two C_3_H_6_ molecules located in one unit cell adopted a quasi-orthogonal arrangement from a static view, giving the dihedral angles of 93.5° for label **1** and 88.3° for label **2** (Figure 3c), respectively. Likewise, for the C_3_H_6_ molecules located at labels **3** and **4**, the geometrical planes also displayed quasi-orthogonal packing models, with dihedral angles of 96.4° for label **3** and 90.5° for label **4** (Figure S7). Such an orthogonal array of C_3_H_6_ molecules would maximize its binding interactions with a polar pore surface and favor its preferential capture.

Subsequently, density functional theory (DFT) optimizations of GCMC derived host–guest structures showed that there were mainly two adsorption sites (termed as site I and site II) for C_3_H_6_-loaded **1** (Figure 3d,e and Table S7), being well located at the unit cell through multiple binding interactions. In detail, for C_3_H_6_-adsorbed **1** in site I, two hydrogen atoms in the methyl group were grasped through forming chummy van der Waals interactions (C–H···π) with adjacent triazole ligands, giving binding distances of 2.46 Å (D1) and 2.86 Å (D2), respectively (Figure 3d). Meanwhile, another hydrogen atom in the propenyl group and the one in the methyl group were confined through forming additional hydrogen bonds with the oxygen atom in the oxalate ion, yielding shorter distances of 2.50 Å (D3) and 2.37 Å (D4). It should be noted that the latent intramolecular interaction between two C_3_H_6_ molecules also favored the C_3_H_6_ adsorption. As visually observed in Figure S8, the static conformation of C_3_H_6_ adsorbed in site I after geometry optimization oriented linearly with its C=C axis along the vertical direction of the propylene plane in site II for yielding intramolecular affinities through forming Lewis acid/base interactions, yielding a binding distance (C^δ−^···H^δ+^) of 2.56 Å for D5 and 2.42 Å for D6 (Figure 3d and Figure S8). Similar with that in site I, other C_3_H_6_ molecule in site II were also confined through strong binding affinities. As seen in Figure 3e, in addition to the mentioned intramolecular interaction (namely D5 and D6, inset in Figure 3d), the hydrogen atoms in the methyl group and methylene group were spatially captured with a combination of the van der Waals effects (distance: 2.59 Å for D1 and 2.48 Å for D2). In addition, other H atoms in the propenyl and methyl groups were well immobilized through hydrogen-bond interactions with oxygen atoms (distance: 2.72 Å for D3 and 2.48 Å for D4; Figure 3e). On the contrary, for C_3_H_8_-loaded **1**, it suggested a weaker binding interaction, although having two adsorption sites (detailed adsorption configurations are available in Figure S9a,b). The theoretical binding energies between the host and guest showed an expected *Q*_st_ order of C_3_H_6_ (**1** (38.9 kJ mol^–1^) > C_3_H_8_ (**1** (26.4 kJ mol^–1^), which was consistent with that obtained from experimental results (38.3 kJ mol^–1^ for C_3_H_6_ and 28.9 kJ mol^–1^ for C_3_H_8_; Figure 2f). Notably, the binding interactions between the gas molecules and framework were modest (van der Waals interaction, hydrogen bonding, and electrostatic interaction in nature), indicating that enriched C_3_H_6_ can then be recovered with high purity during the regeneration step.

To further elucidate the unwonted geometrical conformation and adsorption mechanism of C_3_H_6_ in the **1** skeleton, an in situ PXRD test and Rietveld structural refinements of C_3_H_6_-loaded **1** were carefully analyzed. As shown in Figure S10a, the refined lattice parameters (*a* = 8.9427 Å, *b* = 9.7210 Å, *c* = 9.5827 Å) of C_3_H_6_-loaded **1** seemed to be somewhat larger than that of pristine **1** (*a* = 8.9139 Å, *b* = 9.6932 Å, *c* = 9.4836 Å; Table S3), attributed to C_3_H_6_ occupation in the unit cell of **1**. An intuitional binding scenario (Figure S10b,c) yielded from structural refinements demonstrated that the geometry configurations derived from in situ crystallographic experiments were quasi-consistent with those calculated by theoretical DFT calculations (Figure 3d,e). By comparing the binding distances of the same binding types obtained from experimental and theoretical results, the low relative errors are between 0.0 and 3.1% (Table S8). Such lower relative errors further reveal that the adsorption conformation yielded from crystallographic tests were convincing. In addition, the geometrical planes of two C_3_H_6_ molecules also exhibited an approximate orthogonal adsorption configuration from a static view, with a dihedral angle of ca. 93.1° (Figure S10d), being consistent with that obtained from theoretical values.

Further, in situ Fourier transform infrared (FT-IR) tests were recorded to reveal the potential host–guest interaction between C_3_H_6_ and **1**. As clearly shown in Figure S11a, the peaks located in 1630, 1320, and 1171 cm^–1^ in both activated **1** and C_3_H_6_-loaded **1** were assigned to the C=N adsorption band, symmetric carbonyl stretching, and C–O stretching vibrations in **1** structure.^32−34^ While for C_3_H_6_-loaded **1**, some characteristic peaks associated with the propylene molecule were clearly observed. For example, the emerging peaks located at about 2924 and 1436 cm^–1^ could be attributed to symmetric C–H bending of terminal methyl and methylene motifs in propylene.^35,36^ Also, the peak at around 996 cm^–1^ was the C–C stretching mode in propylene.^37^ Such spectral changes evidently confirmed the fact that propylene molecules could be adsorbed in the **1** structure. We investigated in situ Raman spectra of C_3_H_6_-loaded **1** to carefully analyze the host–guest interaction. As explicated in Figure S11b, the peak concentrated at 3147 cm^–1^ was the stretching mode of the C–H bond in the triazole ring of the **1** structure,^38^ and the other Raman vibration of the triazole ring (i.e., heterocyclic methyl C–H bending) could be observed at 1477 cm^–1^.^38^ In addition, it was also found that there was an emerging peak that appeared at 1624 cm^–1^, corresponding to the C=C stretching vibrations in the propylene molecule. This value was downshifted in comparison with that for gaseous propylene, which was 1640 cm^–1^,^39^ mainly ascribed to the formed binding interaction between propylene and the framework, as demonstrated by Yaghi.^40^ The negative shift to lower frequency could also be visualized for the Zn–N peak (Figure S11b). Obviously, the Zn–N peak of **1** also experienced a downshift behavior upon propylene adsorption on **1**, suggestive of potential adsorption sites between propylene and **1**.

In addition, Hirshfeld surface analysis was used for probing the host–guest interactions and quantifying the binding interaction types. Hirshfeld surface was a novel partitioning of crystal space, affording a unique 3D d_norm_ surface which could further be resolved into a 2D fingerprint plot.^41^ As shown in Figure 3f, it revealed the Hirshfeld surfaces of adsorbed C_3_H_6_ in site I mapped over *d*_norm_, where the larger red spots in the map indicated strong short-range interactions with close contact effects (i.e., hydrogen-bonding) and a negative *d*_norm_ value. White spots corresponded to contacts around the van der Waals separation and with a *d*_norm_ value of zero, and blue spots reflected the long-range binding contact with a positive *d*_norm_ value.^42,43^ Clearly, for C_3_H_6_-loaded **1** at site I, hydrogen-bonding interactions (red spots) and van der Waals effects (white spots) reflected in the map (Figure 3f) were basically consistent with the results obtained by DFT calculations (Figure 3d). Further, the binding types could be quantified by plotting two-dimensional (2D) fingerprint plots. Results showed that van der Waals interaction occupied 48.6% of the total Hirshfeld surface, while hydrogen bonding and intramolecular interaction occupied 33.0 and 18.4%, albeit not being particularly precise. Such consistence also could be found for the C_3_H_6_ molecule adsorbed at site II (Figure S12).

### 3.4 Molecular Dynamics and Diffusion Analysis

Diffusion-driven adsorption behavior, a realistic and significative separation metric in industrial PSA, VSA, and TSA-based nonequilibrium working conditions, needed to be given enough attention. We hereby adopted a molecular dynamics (MD) method to probe the diffusion behavior of guest molecules in channels. During the simulations, the initial configurations for the MD simulations were produced by GCMC simulation; the host framework and the gas molecule were both regarded as rigid. As shown in Figure S13 and Table S9, MD-derived diffusion coefficients of CH_4_/C_2_H_6_/C_2_H_4_/C_3_H_8_/C_3_H_6_ for **1** were calculated as 0.217/0.717/0.517/13.3/258 × 10^–11^ m^2^ s^–1^. Therefore, the obtained diffusion coefficient of C_3_H_6_ (258 × 10^–11^ m^2^ s^–1^) on **1** was much higher than that of the covered UiO-66 analogue (97.8 × 10^–12^ m^2^ s^–1^)^44^ and MAF-23-O (0.82 × 10^–10^ m^2^ s^–1^),^28^ confirming the fast diffusion rate. The obtained diffusion selectivity of C_3_H_6_/C_3_H_8_ was calculated to be 19.4, which was eclipsed compared with that of MAF-23-O (112.3). Intuitional snapshots of the MD results with both the host and guests as rigid suggested that two C_3_H_6_ molecules can be adaptively located in the confined channel of **1** after being steadily confined in the pore pocket (Figure S14b), yet that in **1** seemed to be escaping from the host when entering the aperture of the host framework (Figure S14a). Such transient diffusion trajectories further confirmed the favorable diffusion intension for trapping C_3_H_6_ molecule.

### 3.5 Dynamic Column Breakthrough Experiments

**Figure 4:**
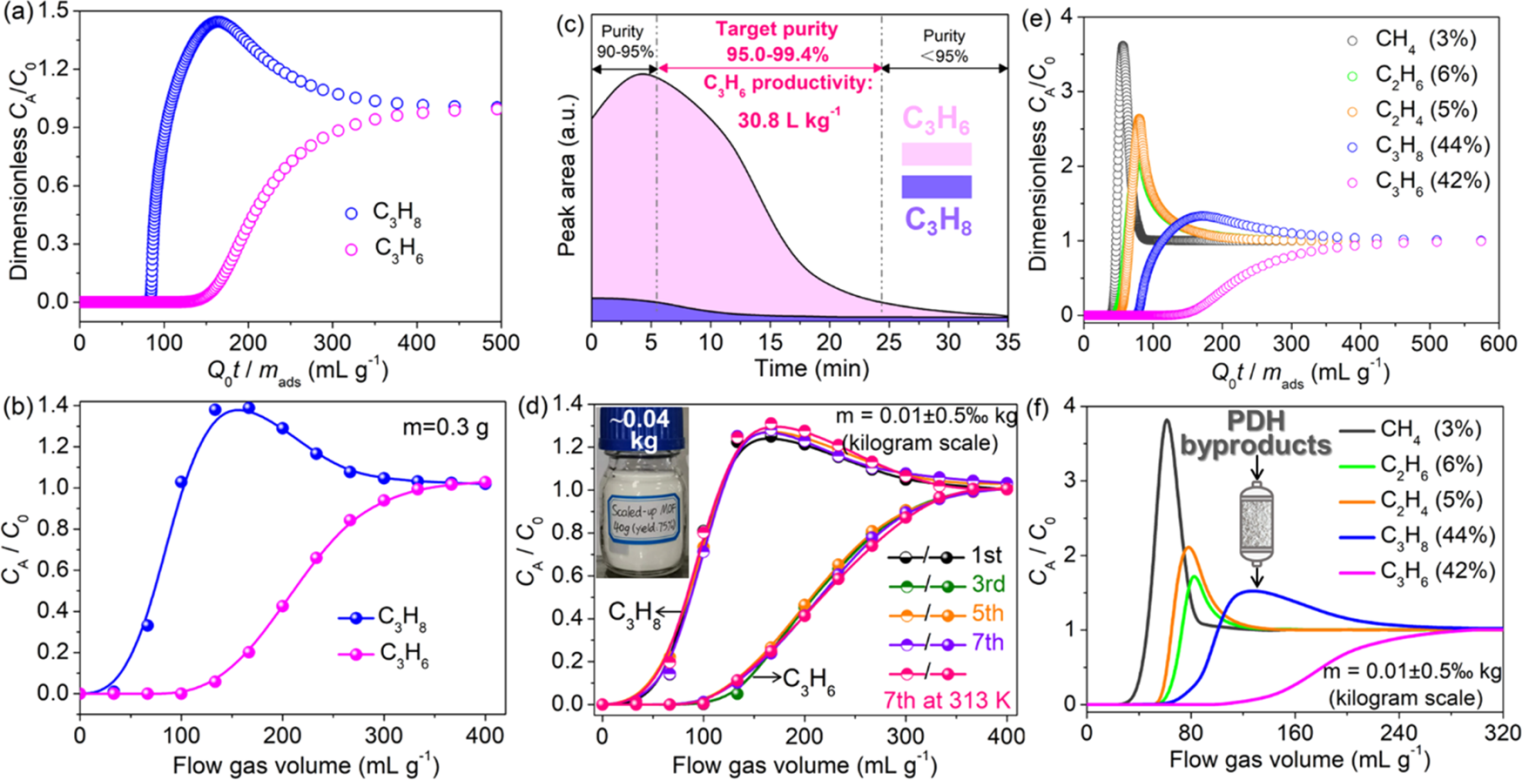
(a) Simulated breakthrough curves of **1** for C_3_H_6_/C_3_H_8_/He (30/30/40, v/v/v) at 298 K. (b) Experimental breakthrough curves of **1** for C_3_H_6_/C_3_H_8_/He (30/30/40, v/v/v) at 298 K and 1 bar, with a flow rate of 2 sccm. (c) Desorption curves of adsorbed C_3_H_6_ and C_3_H_8_ through helium purge, with a flow rate of 5 sccm. (d) Cycling breakthrough experiments of **1** for C_3_H_6_/C_3_H_8_/He (30/30/40, v/v/v) over eight cycles (inset represents the scaled-up production on the kilogram level). (e) Simulated breakthrough curves and (f) experimental breakthrough curves of **1** for quinary CH_4_/C_2_H_4_/C_2_H_6_/C_3_H_6_/C_3_H_8_ (3/5/6/42/44, v/v/v/v/v) mixtures at 298 K and 1 bar.

To further explore separation natures of propylene on **1** from imitated propane dehydrogenation byproducts, transient breakthrough simulations were first probed for C_3_H_6_/C_3_H_8_/He (30/30/40, v/v/v) mixtures in a column adsorption–desorption cycle. Simulation results suggested that C_3_H_6_/C_3_H_8_ mixtures with distinct breakthrough time could be effectively separated (Figure 4a), yielding a higher C_3_H_6_ capture capacity of 51.5 L kg^–1^. These excellent breakthrough results from simulation motivated us to evaluate the separation performance of **1** in the actual separation process. As shown in Figure 4b, C_3_H_8_ first broke through the adsorption bed, while targeted C_3_H_6_ was still captured over a flow gas volume of 100 mL g^–1^. To be noted, the simulated breakthroughs are sharper than those observed experimentally, mainly attributed to the fact that, in the simulations, intracrystalline diffusional influences are ignored.^26^ The captured C_3_H_6_ and C_3_H_8_ uptakes were also calculated to be ca. 52.0 and 2.0 L kg^–1^, giving the outstanding experimental selectivity (or separation factor) of 26. Such a value was much higher than that of other benchmark materials including ZU-36-Ni (19.1),^31^ MAF-23-O (15),^28^ and Co(AIP)(BPY)_0.5_ (2.92)^45^ etc. We also evaluated the captured amount and experimental selectivity with other well-known materials to demonstrate the “trade-off” effects (namely incompatible adsorption capacity and selectivity). As obviously seen in Figure S15, **1** was compatible with adsorption capacity and selectivity, anticipated to be a late-model paradigm for trace C_3_H_6_ purification.

In addition to the capacity and adsorption selectivity mentioned above, the productivity and purity of C_3_H_6_ yielded from desorption operation were also two important metrics to assess the separation performance of adsorbents. Apparently, the outlet concentration of the C_3_H_6_ product was much higher than that of the C_3_H_8_ competitor, and the whole desorption process can be fully desorbed within 35 min (Figure 4c). The obtained C_3_H_6_ purities at different time periods were somewhat different, among which the obtained propylene purity between 5.4 and 24.3 min was up to 99.4%, albeit being somewhat overshadowed with actual requirements (≥99.5%) required by the polymer-grade purity of propylene.^46^ In addition, it could be estimated that about 30.8 L of the C_3_H_6_ product with a serviceable purity of 95.0–99.4% could be accomplished from the equimolar C_3_H_6_/C_3_H_8_ mixtures for 1 kg of activated **1** in a single breakthrough operation. Such higher productivity and purity far exceeded that of KAUST-7 (16.3 L kg^–1^ with 90.0% purity) and Y-abtc (1.3 L kg^–1^ with 90.0% purity), yet was inferior in comparison to the laudable precedent, i.e., JNU-3a (34.2 L kg^–1^ with 99.5% purity) reported so far.^11^ In actual industrial separation units, there is a huge gap between laboratory pilot studies and commercial applications, making mass production of MOFs on a large scale a strong necessity. Here, kilogram scale breakthrough tests were conducted through filling 0.01 ± 0.5‰ kg of activated **1** into the customized column. As revealed in Figure 4d, **1** could still retain the quasi-unchanged separation performance for eight cycles, even in the presence of flow moisture having a certain temperature. The PXRD spectra after eight cycles still kept its intact crystalline structure when immersed in water for 1 week (Figure S16), echoing well with its excellent structural rigidity and stability.

Although **1** suggested efficient C_3_H_6_ separation from C_3_H_6_/C_3_H_8_ binary mixtures, there are tremendous current impediments and challenges in the recovery of valuable propylene from propane dehydrogenation byproducts (typically consisting of ca. 1–3% CH_4_, 0.5–6% C_2_H_6_, 0.2–5% C_2_H_4_, 40–45% C_3_H_8_, and 42–50% C_3_H_6_). Targeting the C_3_H_6_ purification from quinary mixtures could be expected to earn substantial economic benefits. Transient breakthrough simulations were first predicated with the various feed compositions of CH_4_/C_2_H_4_/C_2_H_6_/C_3_H_6_/C_3_H_8_ (3/5/6/42/44, v/v/v/v/v) to assess the universality of **1** for separation of quinary components. As shown in Figure 4e, efficient separation can be accomplished by **1** for quinary mixtures, wherein CH_4_, C_2_H_6_, C_2_H_4_, and C_3_H_8_ occurred first, and then C_3_H_6_ passed through the column after a certain time (τ_break_). Further, the experimental breakthrough tests were tested in the packed column of **1** under the same gas feed ratios under ambient conditions. The breakthrough profiles described in Figure 4f evidently confirmed that **1** could effectively purify C_3_H_6_ from imitative propane dehydrogenation byproducts.

### 3.6 Structural Stability Tests and Costs Evaluation

It is well perceived that structural stability is the first and necessary prerequisite for MOFs to develop from laboratory research to the pilot scale and industry applications. We herein carried out multifaceted investigations on **1**, which had experienced cycling breakthrough experiments, including N_2_ adsorption, single-gas adsorption, and crystallographic tests under variable-temperature conditions etc., to systematically analyze the structural stability of **1**. We first probed the pore geometry changes through N_2_ adsorption at 77 K. As shown in Figure S17a, the N_2_ uptake of **1** still inherited the quasi-unchanged adsorption capacity after cycling breakthrough tests, and the aperture distribution was concentrated at 4.67 Å (inset in Figure S17a), suggesting the unfading ultramicropore nature. In addition, single-gas adsorption isotherms of C_3_H_6_ on **1** yielded a higher capacity of 31.1–35.0/88.5–94.1 cm^3^ cm^–3^ at 0.01/0.1 atm and 298 K after 10 cycles (Figure S17b). Further, the variable-temperature PXRDs of **1** after cycling tests were recorded. As observed in Figure S17c, all of the diffractions all exhibited extremely consistent patterns compared with the theoretical diffractions, indicative of intact structural integrity. The derived top contour plots of variable-temperature PXRD (Figure S17d) evidenced that a main peak shift could hardly be observed, confirming a higher structural rigidity and thermal stability.

Of particular note was that the costs of raw materials should also be taken into account for laboratory-scale synthesis. As visualized in Figure S18 and Table S10, the total costs of raw materials were just $167 for per kilogram of **1** adsorbent, which were much cheaper than other materials, including ZU-36-Ni ($17 399), Fe_2_(dobdc) ($4527), and MCF-57 ($10 073) etc. (Table S10), further reinforcing its potential application for C_3_H_6_ purification. Therefore, the excellent separation performance, steam stability, scalability of production, and cheap costs etc. awarded **1** the prominent potential to purify C_3_H_6_ from PDH byproducts.

## 4 Conclusions

To sum up, an ultramicroporous Zn-MOF with scaled-up production could be easily synthesized using the cost-effective raw materials. An optimized geometry model revealed the ultramicroporous pore conformation for **1**, with a cross-sectional size of 4.27 × 4.60 Å^2^ (19.64 Å^2^). The decent pore aperture was slightly larger than that of the propylene molecule with a minimum cross-section size of 4.16 × 4.65 Å^2^ (19.34 Å^2^) but less than that of propane molecule with minimum cross-section size of 4.20 × 4.80 Å^2^ (20.16 Å^2^), anticipating a diffusion barrier for the highly similar molecules. Static isotherm adsorption suggested that **1** possessed a record-high C_3_H_6_ uptake of 92.4 cm^3^ cm^–3^ at 298 and 0.1 bar, yielding an IAST selectivity of up to 63 among the reported benchmark MOFs. In situ spectroscopies, crystallographic experiments, and modeling demonstrated that two C_3_H_6_ molecules confined in one unit cell were grasped through multiple binding interaction including van der Waals effects, hydrogen bonding, and intraintermolecular interaction. Molecular dynamics showed that **1** possessed a higher diffusion selectivity of 19.4 for C_3_H_6_/C_3_H_8_. Column breakthrough tests demonstrated that about 30.8 L of C_3_H_6_ product with a purity of 95.0–99.4% could be accomplished from the equimolar C_3_H_6_/C_3_H_8_ mixtures for 1 kg of activated **1** in a single breakthrough operation. Such an excellent separation property of propylene on **1** is also validated by the experimental and simulated breakthrough tests of quinary PDH byproducts containing CH_4_/C_2_H_4_/C_2_H_6_/C_3_H_6_/C_3_H_8_ (3/5/6/42/44, v/v/v/v/v). Particularly, structurally stable **1** can be easily synthesized on the kilogram scale using cheap raw materials (only $167 for per kilogram of **1**). The excellent separation performance, steam stability, scalability of production, and cheap costs etc. awarded **1** the prominent potential to purify C_3_H_6_ from PDH byproducts.

## Supporting Information Available

Structural characterizations, spectroscopy tests, modeling details, calculation of separation potential, transient breakthrough simulations, supporting figures and tables (PDF)
