# Supplementary material for: Quasi-Orthogonal Configuration of Propylene within a Scalable Metal–Organic Framework Enables Its Purification from Quinary Propane Dehydrogenation Byproducts
Source: ACS Cent Sci. 2022 Jul 22;8(8):1159–68. doi: 10.1021/acscentsci.2c00554 (PMC9413434; doi:10.1021/acscentsci.2c00554)
Supplement: Supplementary file 1 — oc2c00554_si_001.pdf [file oc2c00554_si_001.pdf]

Supporting information

Quasi-Orthogonal Configuration of Propylene within a Scalable Metal-Organic Framework Enables Its Purification from Quinary Propane Dehydrogenation Byproducts

Peng Hu,[†,1] Jialang Hu,[†,1] Hao Liu,[†] Hao Wang,[†] Jie Zhou,[†]
Rajamani Krishna,[*,§] and Hongbing Ji,[*,†]

[†] Fine Chemical Industry Research Institute, School of Chemistry, Sun Yat-Sen University, Guangzhou, 510275, P.R., China.

[§] Van't Hoff Institute for Molecular Sciences, University of Amsterdam, Park 904, 1098 XH Amsterdam (The Netherlands)

[1] These authors contributed equally to this work.

[*]Email: jihb@mail.sysu.edu.cn; r.krishna@contact.uva.nl

Table of Contents

1. Structural characterizations S3

1.1. Crystal structure analysis and isotherms adsorption S3

1.2. Temperature programmed desorption (TPD) experiments S3

1.3. Calculation of desorption activation energy S3

2. Spectroscopy tests S5

2.1. In situ Fourier transform infrared spectroscopy S5

2.2. In situ Raman spectroscopy test S5

3. Modeling details S6

3.1. Crystal modeling and optimization analysis S6

3.2. Grand Canonical Monte Carlo (GCMC) calculation S6

3.3. Molecular dynamics (MD) simulations S6

4. Calculation of separation potential S7

4.1. Dual Langmuir-Freundlich parameter fits S7

4.2. Calculations of ideal adsorbed solution theory S7

4.3. Calculations of isosteric heat S7

5. Transient breakthrough simulations S8

Supporting figures S10

Supporting tables S29

1. Structural characterizations

1.1. Crystal structure analysis and isotherms adsorption

Powder X-ray diffraction (PXRD) experiment was conducted on the Bruker D8 Advance X-ray diffractometer with Cu Ka emission at room temperature. In situ PXRD patterns were collected at 298 K using a capillary tube packed with the sample, which was firstly evacuated, and then filled with C_3H_6 gas. Then, the Reflex refinements process on C_3H_6-loaded PXRD were carefully conducted through the Reflex Module in Materials Studio *2019* program. Given the fact that a larger number of atoms in one unit cell, the ligand molecule and the gas molecule were both treated as rigid motifs during the refinements process, with the molecule orientation and center of mass freely refined. Finally, the satisfactory *R*-factor and R_{wp} values can be yielded through refining the parameters step by step, including lattice parameters, background, thermal factors, occupancies, profiles, etc.

N_2 adsorption–desorption isotherms were measured through the analyzer (ASAP2460, Micromeritics) at 77 K. The samples were initially degassed under reduced pressure for 12 h at 423 K. Single-gas adsorption experiments of various guests were conducted on the Micromeritic ASAP2020 analyzer. During each experiment, about 150 mg of activated **1** powder was placed in the sample cell and dried for 12 h at 373 K.

1.2. Temperature programmed desorption (TPD) experiments

The TPD experiments were carried out on a gas chromatography workstation at different heating rates from 4 to 8 K min^{-1}. For each operation, 0.02 g of activated **1** sample which had adsorbed guest molecules was packed in a stainless-steel column with an inner diameter of 0.35 cm and a packed length of ca. 0.56 cm. Then the stainless tube was placed in a reaction furnace and heated in the high purity N_2 flow at an initial flow rate of 4 sccm. The desorbed molecule was recorded by using the chromatograph with a TCD detector.

1.3. Calculation of desorption activation energy

Desorption activation energy was an important indicator to assess the binding strength between guest molecule and framework. The TPD curves obtained from chromatograph can be well described by the Polanyi-Wigner equation, which is expressed as Equation 1[1]:

$$r_d = -\frac{d\theta_A}{dt} = k_0\theta_A^m\exp(-E_d RT) \quad (1)$$

where r_d is the desorption rate (mol s^{-1}); θ_A is the fractional surface coverage; k_0 is a constant that depends on the desorption kinetics (s^{-1}); m is the order of the desorption process; E_d is the desorption activation energy of adsorbate (kJ mol^{-1}); R is the gas constant [8.314 J (Kmol)$^{-1}$]. Providing that the desorption process follows first-order kinetics (n = 1), the desorption activation energy can be obtained from Equation 2:

$$\ln\left(\frac{\beta_H}{RT_p^2}\right) = -\left(\frac{E_d}{RT_p}\right) - \ln\left(\frac{E_d}{k_0}\right) \tag{2}$$

where β_H is the heating rate (K min^{-1}) and T_p is the peak desorption temperature (K).

2. Spectroscopy tests

2.1. In situ Fourier transform infrared spectroscopy

The in situ Fourier transform infrared (FTIR) tests were recorded using a Tensor II FTIR spectrometer (Bruker) equipped with an in situ diffuse reflectance cell. Adsorbent was pretreated at 373 K for 6 h (flow rate: 20 sccm) to remove the adsorbed gas impurities and then cooled it to room temperature. After the background signal was collected with the flowing He and then was subtracted. Subsequently, **1** was exposed to propylene with a pressure of 1 atm for 36 h to ensure that the adsorption process has reached equilibrium state. All the spectra were recorded over accumulative 256 scans with a resolution of 4 cm^{-1} in the range of 4000~400 cm^{-1} range.

2.2. In situ Raman spectroscopy test

In-situ Raman spectrum was recorded with a RENISHAW-inVia Raman microscope system (USA). Sample was excited with a focused laser beam (λ = 405 nm), 0.5 mW of output power and 75s of acquisition time were used. First, 0.1 g of **1** sample was placed in heating accessories pure argon gas was bubbled over 20 min to ensure an inert atmosphere. As-synthesized **1** was tested at 298 K and C_3H_6-loaded **1** was activated prior to measurement; activation was implemented in a quartz tube at 373 K for 60 min. Finally, **1** was cooled down to 298 K to get the data. The Raman spectra were recorded in the range of 100~4000 cm^{-1}. Data acquisition were carried out by a computer with Renishaw WiRE Raman software version 2.0.

3. Modeling details

3.1. Crystal modeling and optimization analysis

For isostructural **1**, the Crystallographic data in CIF format have been deposited in the Cambridge Crystallographic Data Centre (CCDC) under deposition numbers: 2084733, which can be obtained free of charge via the link https://www.ccdc.cam.ac.uk/structures/ (or from the Cambridge Crystallographic Data Centre, 12 Union Road, Cambridge CB2 1EZ, U.K.). The initial structure was first optimized in the $Dmol^3$ module, adopting the generalized gradient approximation (GGA) with the Perdew-Burke-Ernzerhof (PBE) functional. The energy, force and displacement convergence criterions were set as 1×10^{-5} Ha, 2×10^{-3} Ha and 3×10^{-4} Å, respectively. To obtain the gas binding energy, an isolated gas molecule placed in a cell unit (with the same cell dimensions as the MOF crystal). The static binding energy (at $T = 0$ K) could be expressed: $E_B = E$ (MOF) + E (gas) - E (MOF + gas).

3.2. Grand Canonical Monte Carlo (GCMC) calculation

The preferential binding conformation between guests and MOF structure were initially searched through GCMC simulations. Note that host framework and the gas molecule were both rigid in GCMC simulations through using Metropolis method, so that the produced the host-guest binding energies were equal to adsorption enthalpies. For all the GCMC simulations, the frameworks and the gas molecules were described by the universal forcefield (UFF). The Mulliken charges and ESP charges, calculated by PDFT, were employed to the framework atoms and guest atoms, respectively. The loading steps, equilibration steps and the production steps were all set to 2.0×10^5 and the temperature was set at 298 K. The cut-off radius was chosen as 15.0 Å for the Lennard-Jones (LJ) potential and the long-range electrostatic interactions were handled by the Ewald & Group summation method.

3.3. Molecular dynamics (MD) simulations

MD simulations were carried out in the Forcite module in Materials studio program. The output is a collection of snapshots in time of the trajectories of the atoms, which can be used in subsequent analysis. The initial host-guest configurations for the MD simulations were produced by GCMC simulations. The host framework and the gas molecule were both regarded as rigid. The constant-volume and temperature (NVT) ensemble were adopted to simulate the dynamic processes. The charges and force field were the same with that for GCMC simulations. The Nose-Hoover thermostat and Berendsen barostat were employed for temperature and pressure control, respectively. The Verlet method with a time step of 5 fs was used to integrate the particle equations of motion, and atomic trajectories were recorded every 5 ps. Besides, the velocity verlet algorithm was used for integration in the MD simulations, which were used to constrain rigid bonds and to integrate the equations of motion. MD simulations were then run for 30 ns (i.e., 1×10^6 steps with a time step of 2 fs)

after 5 ns of equilibration. The configurations were further stored every 2000 timesteps. The electrostatic interactions and the van der Waals interactions were evaluated by the Ewald summation method.

4. Calculation of separation potential

4.1. Dual Langmuir-Freundlich parameter fits

Dual-Langmuir-Freundlich isotherm model was adopted to fit the single-component loadings at 298 K, as shown in Equation 3 and 4 [2].

$$q = N_1\frac{ap^b}{1 + ap^b} + N_2\frac{cp^d}{1 + cp^d} \tag{3}$$

With *T*-dependent parameters *a* and *c*,

$$a = a_0 exp\left(\frac{E_A}{RT}\right); c = c_0 exp\left(\frac{E_B}{RT}\right) \tag{4}$$

Here, *p* is the pressure of the bulk gas at equilibrium with the adsorbed phase (kPa), *q* is the adsorbed amount per mass of adsorbent (mol kg^{-1}), N_1 *and* N_2 is the saturation capacities of site (mol kg^{-1}) of two different binding sites, *a* and *c* (1 kPa^{-1}) is the corresponding adsorption equilibrium constants reflecting the affinity coefficients of sites 1 and 2, and *b* and *d* represent the deviations from an ideal homogeneous surface for site 1 and 2.

4.2. Calculations of ideal adsorbed solution theory

The gas adsorption selectivity at 298 K and 1 bar was calculated using ideal adsorbed solution theory (IAST) on the basis of the single-component adsorption data. The adsorption selectivity for C_3H_6/C_3H_8 separation is defined by Equation 5 [2]:

$$S_{ads} = \frac{q_1/q_2}{p_1/p_2} \tag{5}$$

In above equation, the fitting parameters q_1 and q_2 reflected the molar adsorption in the adsorbed phase in equilibrium with the bulk gas phase with partial p_1 and p_2. In this work, dual-site Langmuir-Freundlich (DSLF) model was applied to fit C_3H_6 and C_3H_8 isotherms.

4.3. Calculations of isosteric heat

The isosteric heat (Q_{st}), being the crucial thermodynamic variable in adsorption process, affording serviceable information about the binding affinity between the adsorbate molecules and the adsorbent surfaces at different coverage. For this sake, the coverage-dependent adsorption enthalpy was evaluated from sorption data profiles measured at 273 and 298 K by adopting virial fitting method. In detail, a Virial-type equation mainly contained parameters a_i and b_i, which were independent of temperature. In the equation, a_i and b_i represent the fitting Virial coefficients, m and n stands for the numbers of coefficients needed to precisely the

isotherms, as shown in Equation 6 [3]:

$$\ln P = \ln n + \frac{1}{T}\sum_{i=0}^{l} a_i n^i + \sum_{j=0}^{m} b_j n^j \tag{6}$$

The value of isosteric heat (Q_{st}) could be achieved by virtue of following Clausius-Clapeyron equation, as defined in Equation 7:

$$Q_{st} = -R\left[\frac{\partial \ln p}{\partial(1/T)}\right]_n = -R\sum_{i=0}^{l} a_i n^i \tag{7}$$

5. Transient breakthrough simulations

The performance of industrial fixed bed adsorbers is dictated by a combination of adsorption selectivity and uptake capacity. Transient breakthrough simulations were carried out for C_3H_6/C_3H_8 (30/30/40, v/v/v) and $CH_4/C_2H_4/C_2H_6/C_3H_6/C_3H_8$ (3/5/6/42/44, v/v/v/v/v) mixtures operating at a total pressure of 100 kPa and T = 298 K, using the methodology described in earlier publications [4-8]. For the adsorber of length, L, cross-sectional area, A, voidage of the packed bed, ε, the volume of MOF is $V_{ads} = LA(1-\varepsilon)$ m^3. If ρ is the crystal framework density, the mass of adsorbent in the bed is $m_{ads} = (1-\varepsilon) \times (L \text{ m}) \times (A \text{ m}^2) \times (\rho \text{ kg m}^{-3})$ kg. The breakthrough simulations are presented in terms of the following parameter, as shown in Equation 8:

$$\frac{(Q_0 = \text{ flow rate at inlet mL min}^{-1}) \times (\text{time in minutes})}{(\text{g MOF packed in tube})} = \frac{Q_0 t}{m_{ads}} = \text{mL g}^{-1} \tag{8}$$

The breakthrough simulations demonstrate the potential of producing product gas C_3H_6 of required purity during the interval $\Delta\tau$.

Notation

a	Langmuir-Freundlich constant for species i at adsorption site A, $Pa^{-\nu_{iA}}$
c	Langmuir-Freundlich constant for species i at adsorption site A, $Pa^{-\nu_{iB}}$
E	Energy parameter, J mol^{-1}
Q_{st}	Isosteric heat of adsorption, J mol^{-1}
c_i	molar concentration of species i in gas mixture, mol m^{-3}
c_{i0}	molar concentration of species i in gas mixture at inlet to adsorber, mol m^{-3}
t	Time, s
T	Absolute temperature, K

Greek letters

ε voidage of packed bed, dimensionless

ν Freundlich exponent, dimensionless

ρ crystal framework density, kg m^{-3}

Supporting figures

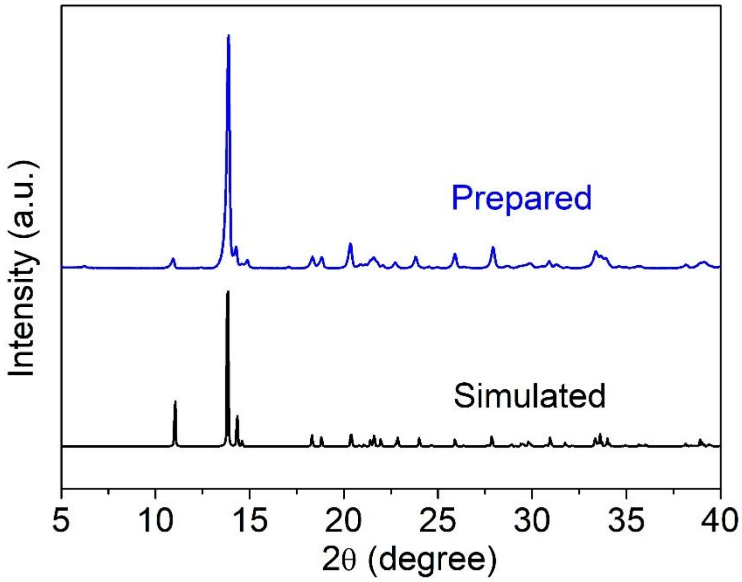

Figure S1. Powder x-ray patterns of **1** simulated from single-crystal topology and obtained experimentally.

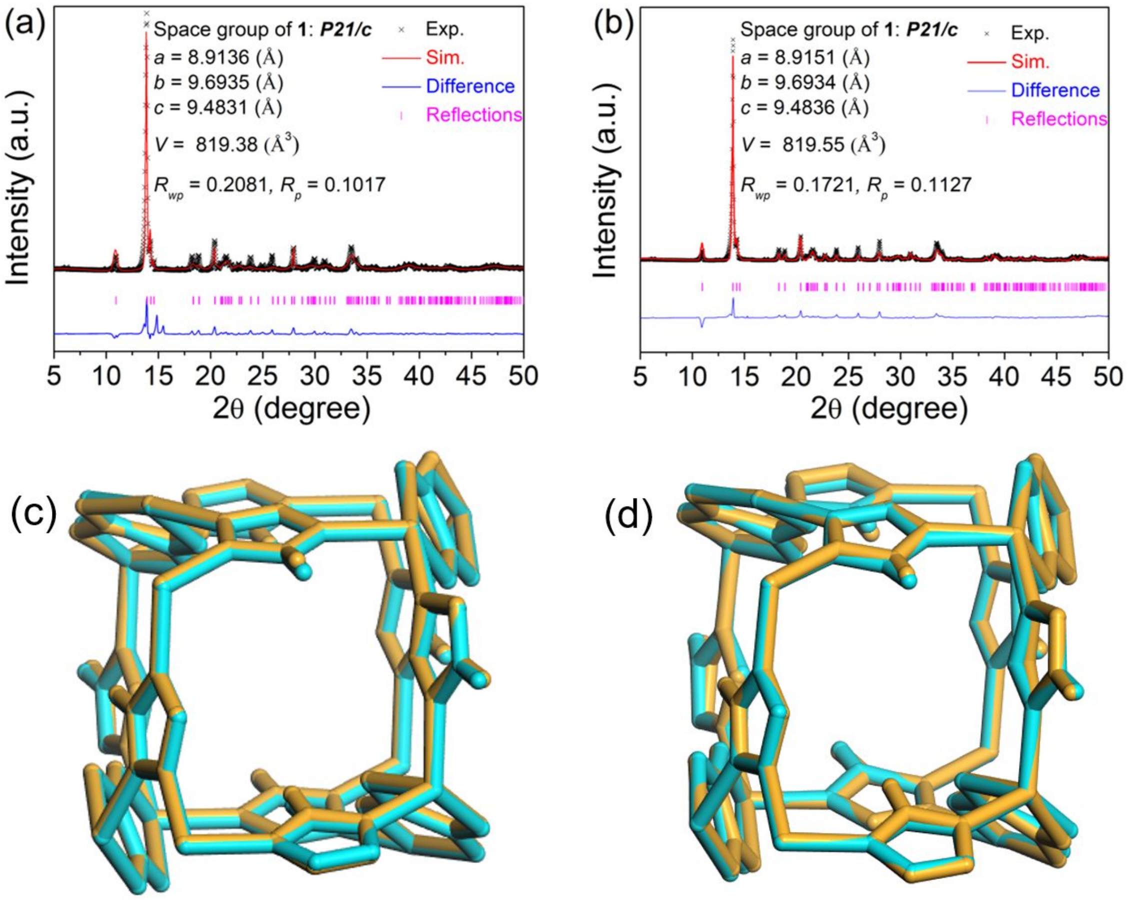

Figure S2. Rietveld structural refinements of **1** recorded at (a) 298 K and (b) 373 K; (c-d) refers to the conformational comparisons of **1** between pristine model structure (orange) and refined structure (turquoise) after heating tests at 298 and 373 K, respectively.

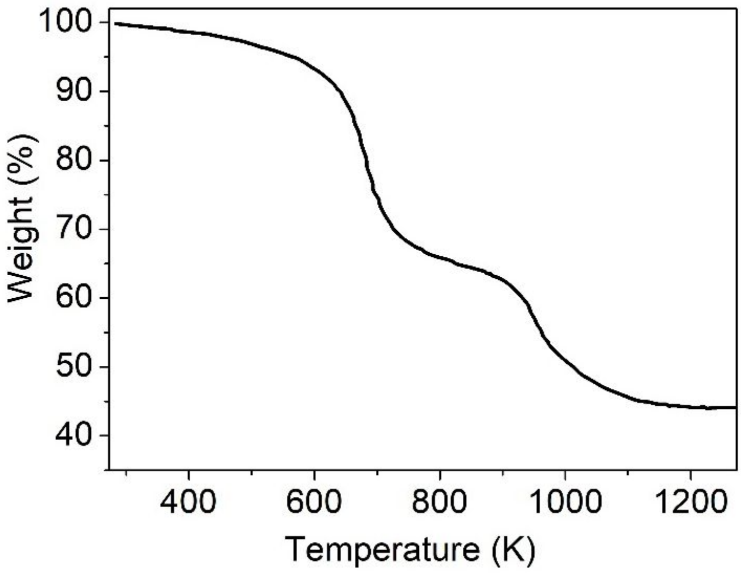

Figure S3. TGA of **1** under air atmosphere.

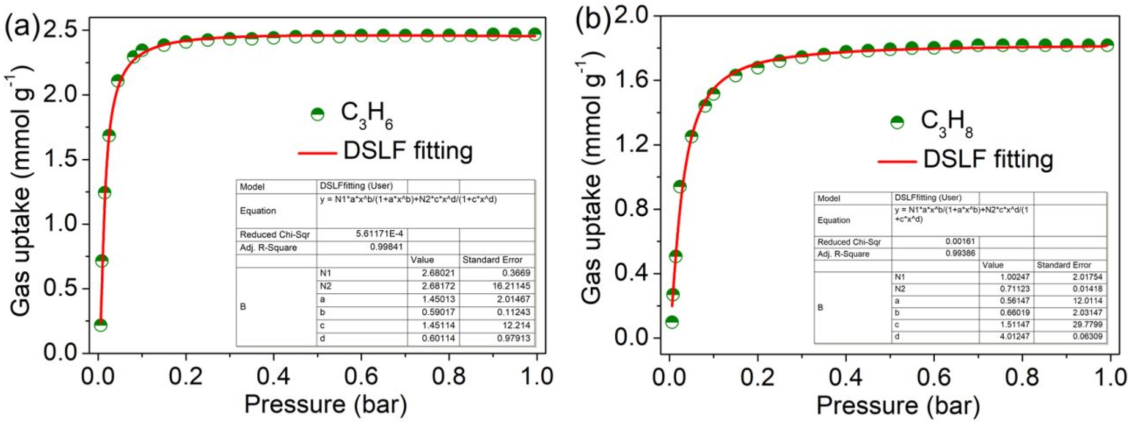

Figure S4. Isotherm fitting of (a) C_3H_6 and (b) C_3H_8 over **1** at 298 K and 1 bar.

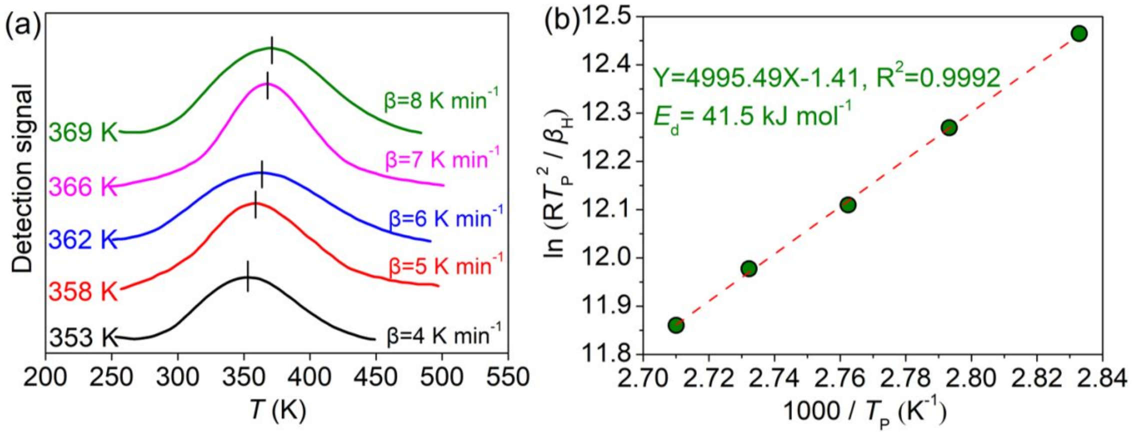

Figure S5. (a) TPD of C_3H_6 on **1** at different heating rates from 4 ~ 8 K min^{-1}; (b) Linear dependence between $-\ln\left(\frac{\beta_H}{RT_p^2}\right)$ and 1000/T_P for TPD of C_3H_6 on **1**.

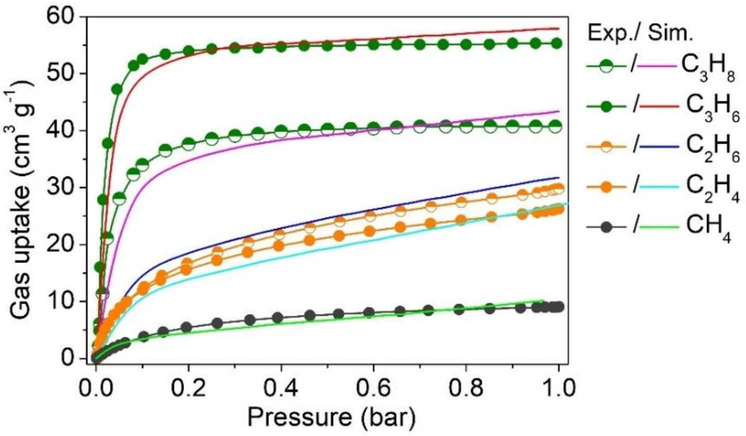

Figure S6. Experimental (dots) and simulated (line) adsorption isotherms of various guests on activated **1** at 298 K and 1 bar.

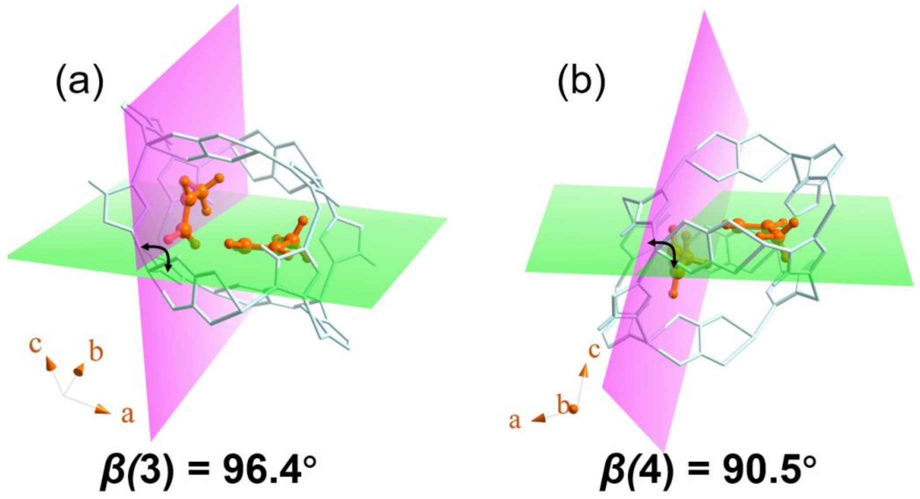

Figure S7. Visualized planes of guest molecule yielded from Figure 3b (**3** and **4**, marking with red color in Figure 3b) and created by three C atoms on a molecule (The atoms in guest molecules are highlighted with orange and other atoms in MOF structure are highlighted with light turquoise for clarity)

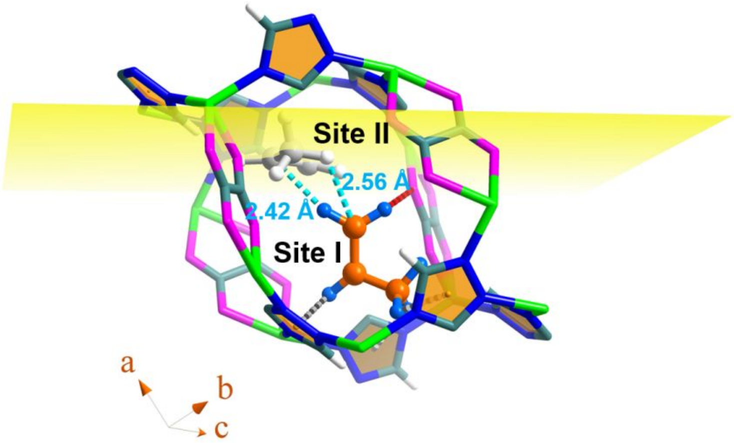

Figure S8. Visualized guest molecule planes created by the three C atoms on a molecule.

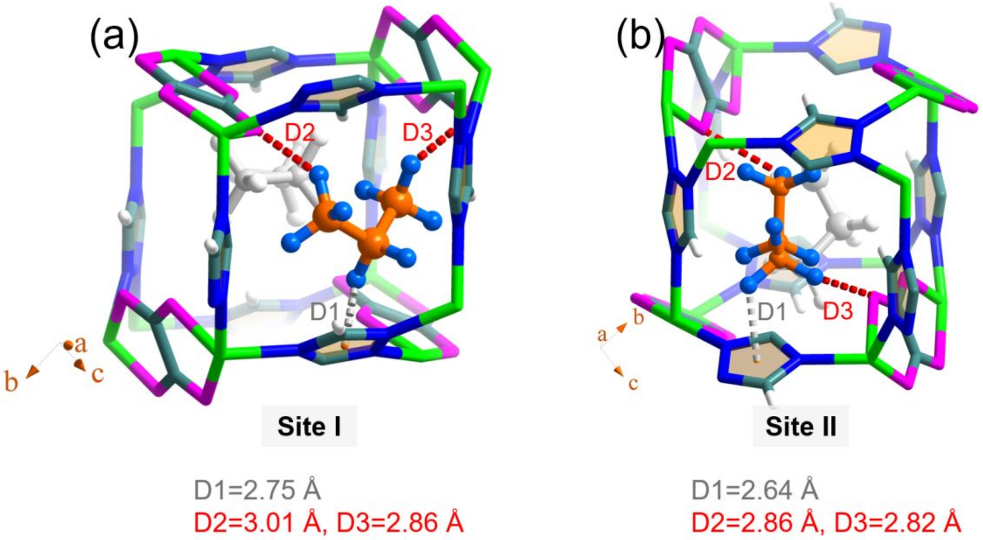

Figure S9. (a) DFT calculated adsorption conformation of C_3H_8-loaded **1** in **Site I** and (b) DFT calculated adsorption conformation of C_3H_8-loaded **1** in **Site II**. (Note that the binding types are colored with gray and red, corresponding to van der Waals interaction and hydrogen-bonding; Color modes: H in ligands, white; H in guest molecule, light blue; Zn, bright green; O, pink; N, blue; C, sea green)

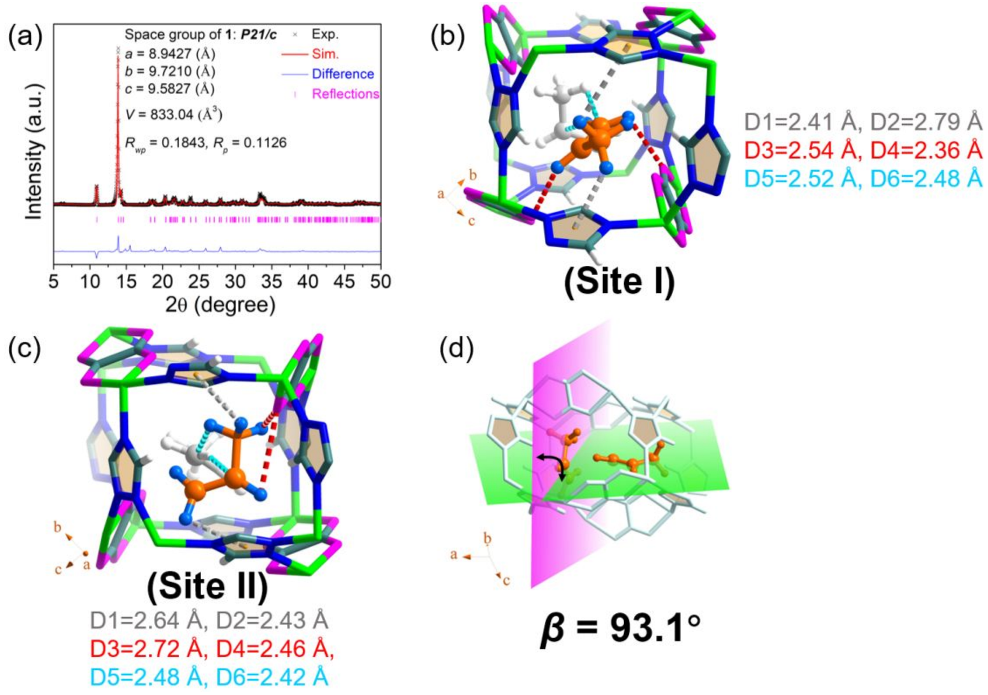

Figure S10. (a) Experimental in situ PXRD pattern (black dots) and refined XRD spectra (red line) of C_3H_6-loaded **1;** Refined geometry conformation of (b) C_3H_6-loaded **1** in **Site I** and (c) C_3H_6-loaded **1** in **Site II**; (d) Visualized guest molecule planes yielded from (b) created by the three C atoms on a molecule.

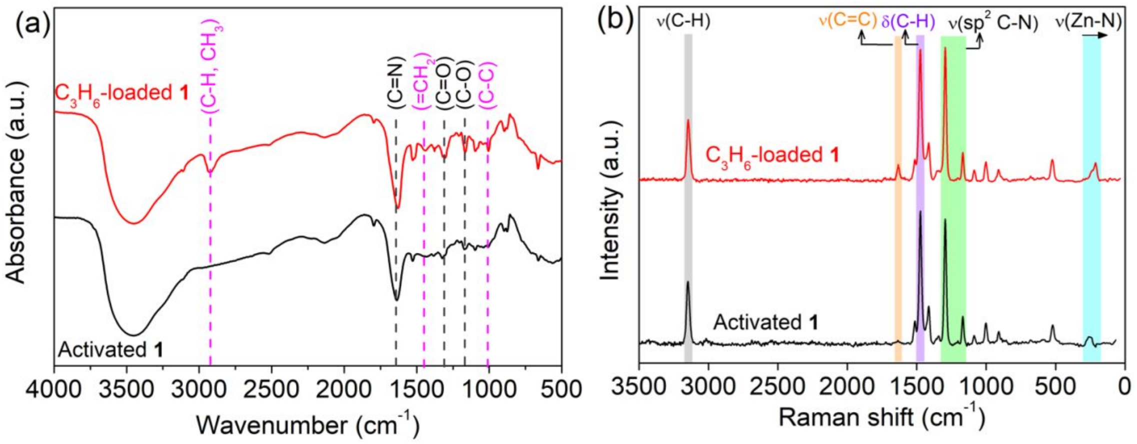

Figure S11. (a) In situ FTIR spectra of activated **1** (black) and C_3H_6-loaded **1** (red) in the wavelength number of 500~4000 cm^{-1}; (b) In situ Raman spectra of activated **1** (black) and C_3H_6-loaded **1** (red), collected with a 405 nm laser.

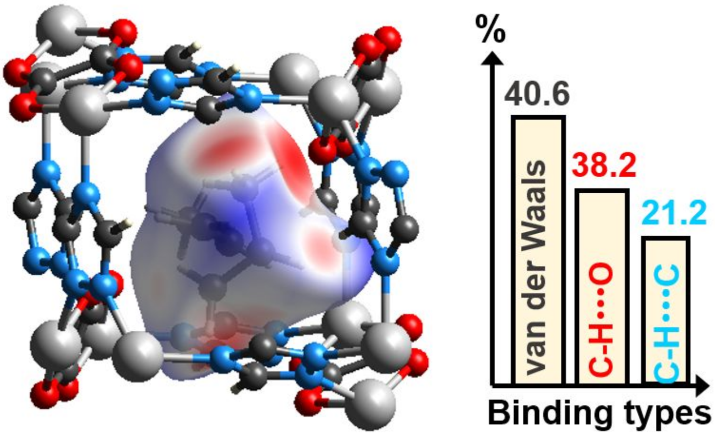

Figure S12. Hirshfeld surface (de) displaying host-guest interactions in **Site II** of C_3H_6-loaded **1** topology.

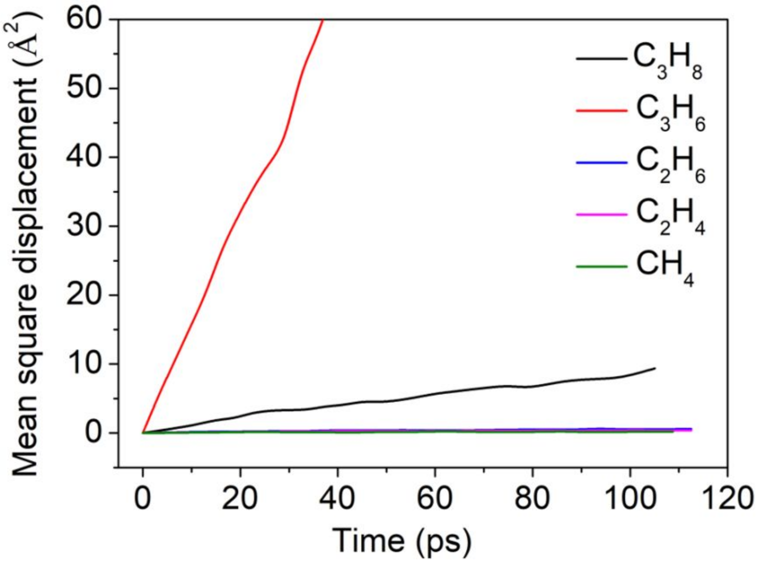

Figure S13. MD-derived self-diffusion rates of various guests in **1**.

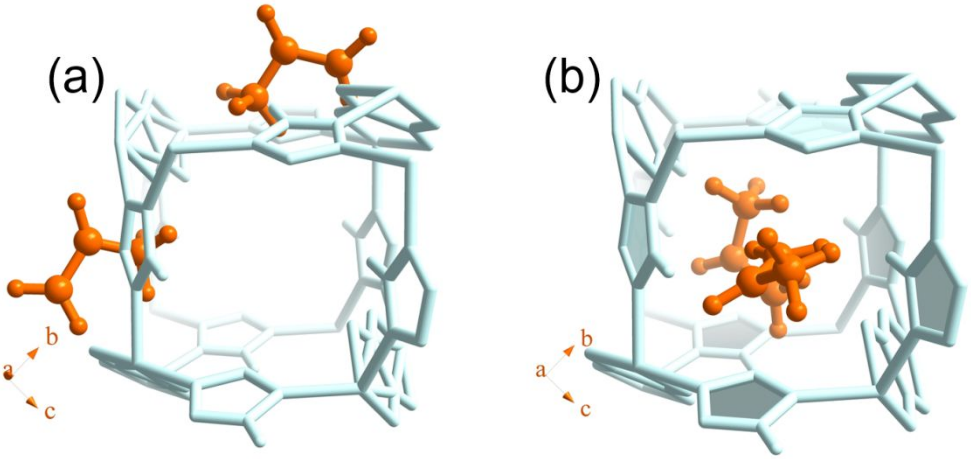

Figure S14. Selected snapshots for MD simulated C_3H_6 adsorption process in **1**: (a) entering aperture of the host framework and (b) staying in the cavity of the host framework with a stable geometry configuration.

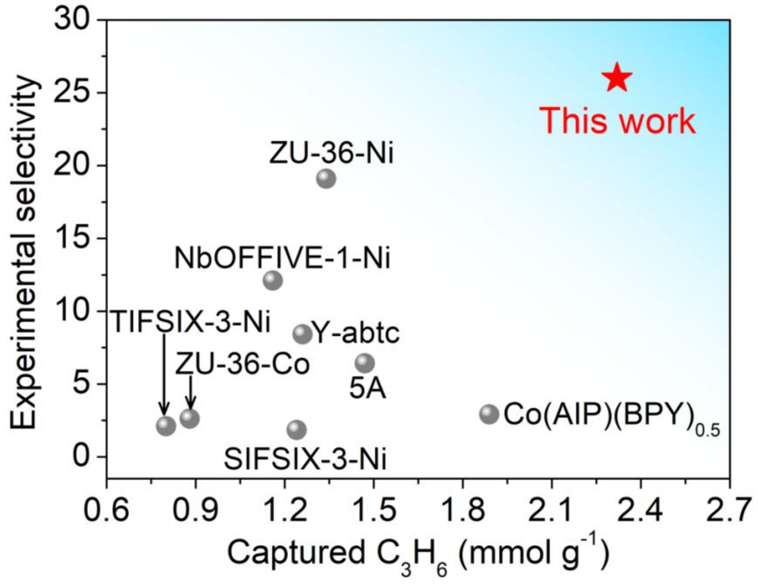

Figure S15. Comparisons of experimental selectivity and C_3H_6 uptake obtained from breakthrough curves over **1** and other benchmark materials.

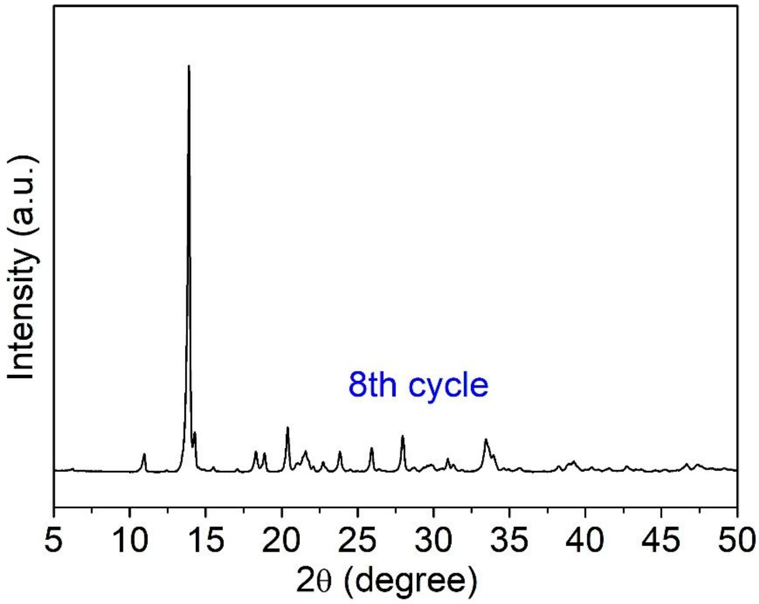

Figure S16. PXRD pattern of **1** after 8th cycle and further immersed in water for one week.

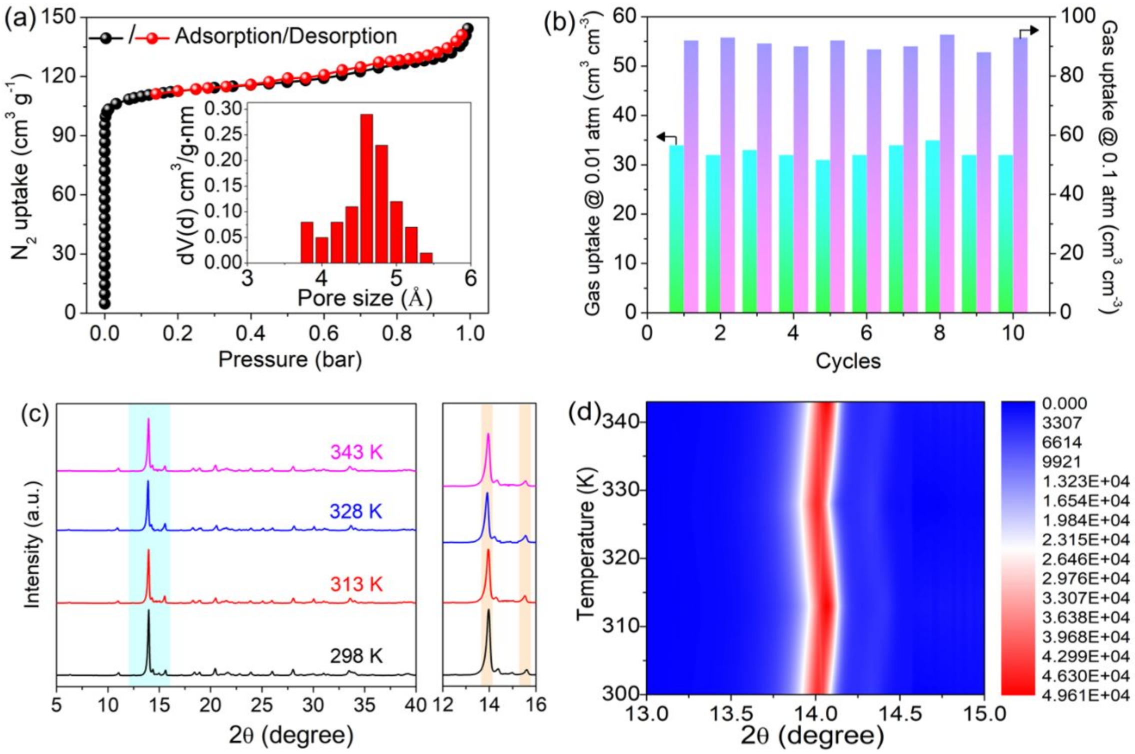

Figure S17. (a) N_2 uptake of **1** after cycling breakthrough tests; (b) Comparison of static C_3H_6 uptake at 0.01/0.1 atm at 298 K after ten cycles; (c) Variable-temperature PXRD of **1** at the temperature of 298 ~ 343 K; (d) Top contour plots of variable-temperature PXRD patters on **1** collected from 298 ~ 343 K in a top view.

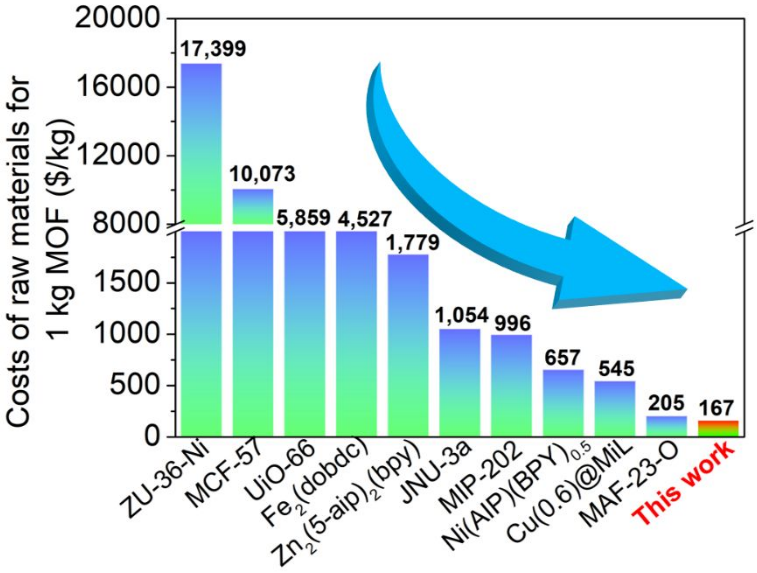

Figure S18. Comparisons of costs of raw materials for **1** and other advanced MOFs.

(For unified comparison, the referenced packages for raw materials and reagents were 100 g and 25 L, respectively)

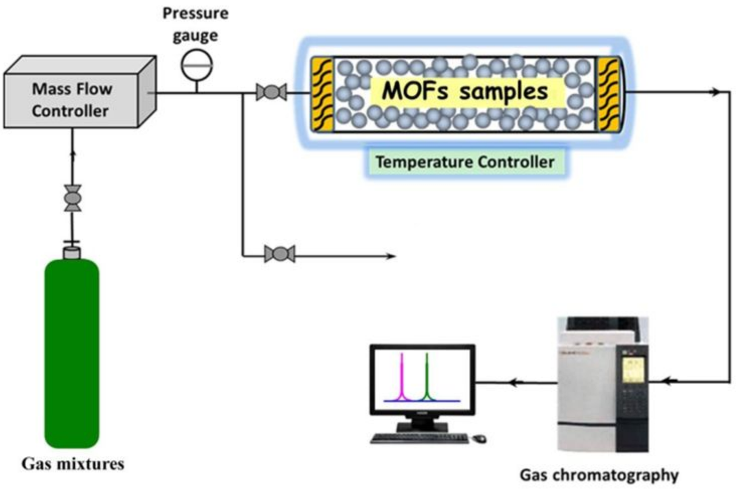

Figure S19. Schematic illustration of the apparatus for the breakthrough tests.

Supporting tables

Table S1. Physical properties of gas molecules used in this work.

Molecule	Kinetic diameter [a] (Å)	Three-dimensional molecule size [b] (Å^3)	Polarizability (× 10^{-25} cm^3)	Dipole moment (× 10^{-18} esu.cm)	Boiling point [c] (K)
CH_4	3.80	3.9 × 4.0 × 4.2	25.93	0	111.5
C_2H_4	4.16	3.4 × 4.2 × 4.9	42.7	0	169.3
C_2H_6	4.44	3.9 × 4.2 × 5.1	44.3-44.7	0	184.4
C_3H_6	4.7	4.16 × 4.65 × 6.45	62.6	0.366	225.5
C_3H_8	5.1	4.20 × 4.80 × 6.80	63.7	0.084	231.0

[a] Kinetic diameter refers to the shortest distance of two colliding identical molecules whose kinetic energies are equal to zero, in which the molecular shape is simplified as a sphere. The value is calculated from the experimental virial coefficients of the gas by assuming that the intermolecular interactions follow the Lenard-Jones potential, but the accurate virial coefficients are very difficult to measure. Therefore, the kinetic diameter of complicated molecules like C_2 differ a lot.

[b] Calculated from the atomic positions from the molecular geometries derived by DFT optimization and corresponding van der Waals radii (C/H/O: 1.7/1.2/1.5 Å).

[c] refers to the value at 298 K.

Table S2. List of atomic positions for Zn-MOF (denoted as **1)** model obtained from crystallography files.

NO.	Element	Symbol	x/a	x/b	x/c
1	Zn	Zn1	0.17588	0.05771	0.43679
2	N	N1	0.0308	-0.1108	0.3683
3	N	N2	-0.0922	-0.1475	0.4100
4	N	N3	-0.0992	-0.2914	0.2259
5	O	O1	0.4098	0.0761	0.6102
6	O	O2	0.6753	0.0307	0.6732
7	C	C1	0.0215	-0.1983	0.2588
8	H	H1	0.0932	-0.1955	0.2086
9	C	C2	-0.1655	-0.2554	0.3232
10	H	H2	-0.2559	-0.3029	0.3289
11	C	C3	0.5248	0.0308	0.5815

Table S3. Crystallographic parameters and refinement details of experimental and theoretical models.

Crystals	**1[#]**	**1**	**1@298 K**	**1@373 K**
Formula weight	195.72	195.76	195.72	196.75
Crystal system	monoclinic	monoclinic	monoclinic	monoclinic
Space group	***P21/c***	***P21/c***	***P21/c***	***P21/c***
a (Å)	8.9138	8.9139	8.9136	8.9151
b (Å)	9.6934	9.6932	9.6935	9.6934
c (Å)	9.4839	9.4836	9.4831	9.4836
Cell volume ($Å^3$)	819.46	819.42	819.38	819.55
Calc. density (g cm^{-3})	1.762	1.760	1.762	1.763
R_P (%)	4.17	9.48	10.17	11.27
R_{WP} (%)	10.24	16.11	20.81	17.21

[#] represent the values derived from optimized crystal model.

Table S4. Summary of the adsorption capacity, uptake ratio, selectivities and heat of adsorption data for C_3H_6 and C_3H_8 in various propylene-based MOF adsorbents.

Adsorbents	C_3H_6 uptake [a] ($cm^3\ cm^{-3}$)		IAST [b] (50/50, v/v)		Q_{st} (C_3H_6)[c] (kJ mol^{-1})	**Ref.**
	C_3H_6	C_3H_8	0.1 bar	1 bar		
**1**	**92.4**	**59.7**	**63.0**	**107.0**	**38.2** [d]	This Work
UiO-66-CF_3	35.2	22.2	82.1	103.0	38.1 [d]	9
MAF-23-O	35.0	18.5	8.9	8.9	54.0 [d]	10
SIFSIX-2-Cu-i	30.5	9.5	4.2	5.1	35.8 [d]	11
Zn_2(5-aip)$_2$(bpy)	36.2	4.0	14.0	20	46.0 [d]	12
CPL-1	5.4	3.8	N.R. [g]	N.R. [g]	N.R. [g]	13
MIL-100(Fe)	40.9	25.8	3.8	2.0	N.R.	14
ZU-36-Co	41.4	8.3	9.5	15	38.0 [d]	15
GeFSIX-2-Cu-i	32.2	13.7	3.8	4.1	36.2 [d]	16
HOF-16a	52.3	33.0	2.0	5.4	30.2 [e]	17
JNU-3a	14.4	7.5	2.7	513	29.3 [f]	18

[a] refers to adsorption uptake at 298 K and 0.1 bar.

[b] IAST selectivity. These values are only for the qualitative comparison purpose.

[c] Q_{st} values at low surface coverage.

[d] refers to Q_{st} was obtained through the virial method.

[e] refers to Q_{st} was obtained through the Clausius-Clapeyron equation.

[f] refers to Q_{st} was obtained through differential scanning calorimetry.

[g] N.R. represent not reported.

Table S5. Dual-site Langmuir-Freundlich parameters fits for C_3H_6 and C_3H_8 of **1**.

		Site A			Site B		
		N_1	a	b	N_2	c	d
		mol kg^{-1}	Pa^{-vA}	dimensionless	mol kg^{-1}	Pa^{-vA}	dimensionless
**1**	C_3H_6	2.6802	1.4501	0.5902	2.6817	1.4511	0.6011
	C_3H_8	1.0025	0.5615	0.6602	0.7112	1.5115	4.0125

Table S6. The parameters of virial equation for various gas adsorption isotherms on **1**.

Gas	a_0	a_1	a_2	a_3	a_4	a_5	a_6	a_7	b_0	b_1	b_2	R^2
C_3H_8	-3478	401	-882	2001	-1197	2808	-3297	206	18.4	0.017	-0.273	0.9945
C_3H_6	-4608	1468	406	-2808	349	916	-1995	3078	5.78	0.210	-1.27	0.9987
C_2H_6	-2708	822	-1080	2104	204	2747	-3107	76	10.2	-0.004	1.28	0.9889
C_2H_4	-2508	302	-907	1576	198	2574	-2479	128	8.00	0.297	-5.37	0.9967
CH_4	-1008	22	-1026	1508	174	2875	-2439	1022	26.4	0.087	-1.87	0.9937

Table S7. List of atomic positions for C_3H_6-loaded **1** obtained from DFT calculations.

Atom	x/a	x/b	x/c	Atom	x/a	x/b	x/c
O1	0.53007	0.52537	0.2966	O28	0.55843	0.34357	0.55773
O2	0.44157	0.51023	0.2756	C29	0.3405	0.60057	0.4196
C3	0.49173	0.51027	0.30617	H30	0.3644	0.6015	0.40287
Zn4	0.39196	0.48076	0.31226	C31	0.6115	0.58153	0.44107
Zn5	0.60804	0.3141	0.52107	C32	0.50827	0.3436	0.52717
O6	0.53007	0.30797	0.46327	Zn33	0.60804	0.51924	0.3544
O7	0.44157	0.3231	0.44227	N34	0.6564	0.46307	0.37723
C8	0.49173	0.32307	0.47283	N35	0.36407	0.45083	0.36333
N9	0.3026	0.6175	0.47	N36	0.3664	0.40287	0.4247
N10	0.30027	0.56953	0.40863	O37	0.53007	0.52537	0.62993
C11	0.27817	0.58153	0.44107	O38	0.44157	0.51023	0.60893
Zn12	0.27471	0.51924	0.3544	C39	0.6595	0.4339	0.41373
N13	0.32307	0.46307	0.37723	H40	0.6356	0.43483	0.43047
C14	0.32617	0.4339	0.41373	C41	0.3885	0.41487	0.39227
H15	0.30227	0.43483	0.43047	C42	0.49173	0.51027	0.6395
Zn16	0.27471	0.64743	0.52107	Zn43	0.60804	0.64743	0.52107
N17	0.32307	0.37027	0.5439	N44	0.6564	0.37027	0.5439
C18	0.32617	0.39943	0.5804	N45	0.36407	0.3825	0.53
H19	0.30227	0.3985	0.59713	N46	0.3664	0.43047	0.59137
N20	0.3026	0.54917	0.63667	O47	0.53007	0.6413	0.46327
N21	0.30027	0.59713	0.5753	O48	0.44157	0.65643	0.44227
C22	0.27817	0.58513	0.60773	C49	0.6595	0.39943	0.5804
Zn23	0.39196	0.35257	0.47893	H50	0.6356	0.3985	0.59713
N24	0.3436	0.62973	0.4561	C51	0.3885	0.41847	0.55893
N25	0.63593	0.6175	0.47	C52	0.49173	0.6564	0.47283
N26	0.6336	0.56953	0.40863	Zn53	0.39196	0.48076	0.6456
O27	0.46993	0.3587	0.53673	N54	0.3436	0.53693	0.62277
N55	0.63593	0.54917	0.63667	O83	0.55843	0.48977	0.7244
N56	0.6336	0.59713	0.5753	C84	0.50827	0.48973	0.69383
O57	0.46993	0.47463	0.37007	C85	0.38046	0.49996	0.51523
O58	0.55843	0.48977	0.39107	C86	0.4212	0.46972	0.5039
C59	0.3405	0.5661	0.58627	C87	0.4732	0.49803	0.51263
H60	0.3644	0.56517	0.56953	H88	0.3669	0.48094	0.54277
C61	0.6115	0.58513	0.60773	H89	0.34247	0.50784	0.47708

C62	0.50827	0.48973	0.3605	H90	0.40184	0.53348	0.53423
Zn63	0.72529	0.35257	0.47893	H91	0.41015	0.43244	0.49121
N64	0.67693	0.62973	0.4561	H92	0.5013	0.48656	0.49411
C65	0.67383	0.60057	0.4196	H93	0.48358	0.53047	0.5376
H66	0.69773	0.6015	0.40287	C94	0.56762	0.50957	0.46511
N67	0.6974	0.45083	0.36333	C95	0.58889	0.46427	0.49534
N68	0.69973	0.40287	0.4247	C96	0.58856	0.4681	0.54919
C69	0.72183	0.41487	0.39227	H97	0.53398	0.50175	0.42424
N70	0.6974	0.3825	0.53	H98	0.60353	0.5283	0.46206
N71	0.69973	0.43047	0.59137	H99	0.5496	0.53221	0.48667
C72	0.72183	0.41847	0.55893	H100	0.60179	0.43466	0.47675
Zn73	0.72529	0.48076	0.6456	H101	0.61524	0.44389	0.58214
N74	0.67693	0.53693	0.62277	H102	0.56162	0.49515	0.55609
C75	0.67383	0.5661	0.58627				
H76	0.69773	0.56517	0.56953				
Zn77	0.39196	0.6859	0.47893				
O78	0.46993	0.69203	0.53673				
O79	0.55843	0.6769	0.55773				
C80	0.50827	0.67693	0.52717				
Zn81	0.60804	0.51924	0.68774				
O82	0.46993	0.47463	0.7034				

Table S8. Comparisons of binding distances obtained from DFT calculations and in situ PXRD tests.

Binding types	Binding Sites	Distance [a] (Å)	Distance [b] (Å)	Relative error [c] (%)
van der Waals interaction	**Site I**, D1	2.46	2.41	2.0
van der Waals interaction	**Site I**, D2	2.86	2.79	2.4
hydrogen-bonding	**Site I**, D3	2.50	2.54	1.6
hydrogen-bonding	**Site I**, D4	2.37	2.36	0.4
intramolecular interaction	**Site I**, D5	2.56	2.52	1.6
intramolecular interaction	**Site I**, D6	2.42	2.48	2.5
van der Waals interaction	**Site II**, D1	2.59	2.64	1.9
van der Waals interaction	**Site II**, D2	2.48	2.43	2.0
hydrogen-bonding	**Site II**, D3	2.72	2.72	0.0
hydrogen-bonding	**Site II**, D4	2.48	2.46	0.8
intramolecular interaction	**Site II**, D5	2.56	2.48	3.1
intramolecular interaction	**Site II**, D6	2.42	2.42	0.0

[a] represent the binding distance was obtained from DFT calculations

[b] represent the binding distance was obtained from in situ PXRD tests

[c]relative error (absolute value) was calculated based on the equation: $[\frac{\text{Distance}^{b} - \text{Distance}^{a}}{\text{Distance}^{a}} \times 100\%]$

Table S9. Comparison of simulated diffusion parameters for diffusivity.

Adsorbates	Fitting K value ($\times10^{-3}$)	D_M ($\times10^{-12}$ m^2 s^{-1})
C_3H_8	80	133
C_3H_6	1550	2580
C_2H_6	4.3	7.17
C_2H_4	3.1	5.17
CH_4	1.3	2.17

Table S10. Estimated raw material cost for the preparation of C_3H_6-selective adsorbents.

Material	Main raw chemicals	Amount of raw(g) per g product	Label[a]	Price ($)[b]	TCPG[c] ($/kg)	Ref.
**1**	zinc oxalate dihydrate	0.9041 g	Z303988-100 g	8.49	167	This work
	1,2,4-triazole	0.6849 g	T100645-100 g	7.15		
	methanol	9.041 mL	M116115-25 L	102.96		
	ethanol	1.018 mL	E118433-25 L	104.15		
ZU-36-Ni*	nickel tetrafluoroborate hexahydrate	1.199 g	N189039-100 g	65.71	17,399	15
	ammonium hexafluorogermanate	0.7846 g	A167837-5 g	99.68		
	pyrazine	3.523 g	P109613-100 g	26.37		
	water	7.046 mL	W119424-25 L	42.17		
	methanol	7.046 mL	M116115-25 L	102.96		
MAF-23-O	zinc hydroxide	0.3247 g	Z274616-100 g	19.22	205	19
	bis(5-methyl-1H -1,2,4-triazol-3-yl)methane	0.5779 g	A107218-100 g	10.13		
	aqueous ammonia (25%)	12.99 mL	A359072-4 L	19.22		
	water	12.99 mL	W119424-25 L	42.17		
Zn_2(5-aip)$_2$(bpy)	zinc nitrate hexahydrate	1.716 g	Z111703-100 g	25.18	1,880	20
	5-aminoisophthalic acid	0.5228 g	A107450-100 g	16.99		
	4,4'-bipyridine	0.4526 g	B105217-100 g	47.83		
	DMF	117.1 mL	D111999-25L	208.45		
	water	39.02 mL	W119424-25 L	42.17		
UiO-66*	zirconium(IV) chloride	1.207 g	Z109460-100 g	65.41	5,859	9
	terephthalic acid	0.7742 g	P108506-100 g	7.60		
	m-(trifluoromethyl) benzoic acid	0.1055 g	T107270-100 g	22.80		
	DMF	598.1 mL	D111999-25 L	208.45		
JNU-3a	cobalt nitrate hexahydrate	0.7275 g	C112729-100 g	8.34	1,055	18
	5-(3-methyl-5-	0.8100 g	--	---		

	(pyridin-4-yl)-4H-1,2,4-triazol-4-yl)-1,3-benzenedicarboxylic acid					
	methanol	75.00 mL	M116115-25 L	102.96		
	DMA	75.00 mL	D108098-10 L	91.34		
$Ni(AIP)(BPY)_{0.5}$*	nickel acetate tetrahydrate	1.118 g	N112914-100 g	3.13	657	21
	5-aminoisophthalic acid	0.8142 g	A107450-100 g	16.99		
	4,4′-bipyridine	0.3505 g	B105217-100 g	47.83		
	water	44.93 mL	W119424-25 L	42.17		
	methanol	58.41 mL	M116115-25 L	102.96		
MIP-202	zirconium(IV) chloride	1.334 g	Z109460-100 g	65.41	996	22
	L-aspartic acid	1.624 g	A108860-100 g	6.41		
	water	11.60 mL	W119424-25 L	42.17		
MCF-57	cobalt nitrate hexahydrate	0.8595 g	C112729-100 g	8.34	10,073	23
	3-(3-methylpyridin-4-yl)benzoic acid	1.254 g	B176426-100 g	692.55		
	DMA	117.7 mL	D108098-10 L	91.34		
	methanol	58.87 mL	M116115-25 L	102.96		
Cu(0.6)@MIL-100(Fe)*	iron powder	0.2069 g	I116359-100 g	0.92	545	14
	copper(II) chloride	0.3750 g	C106774-100 g	9.54		
	trimethyl 1,3,5-benzenetricarboxylate	0.6259 g	T137552-100 g	73.76		
	nitric acid	0.1490 mL	N116240-500 mL	23.39		
	hydrofluoric acid	0.3222 mL	H278731-2.5 L	12.22		
	water	18.47 mL	W119424-25 L	42.17		
$Fe_2(dobdc)$	anhydrous ferrous chloride	0.5500 g	I106504-100 g	506.45	4,527	24
	2,5-dioxido-1,4-benzodicarboxylate	0.3050 g	D134233-100 g	136.93		
	DMF	150.0 mL	D111999-25 L	208.45		
	methanol	18.00 mL	M116115-25 L	102.96		

a. The prices are based on Aladdin (https://www.aladdin-e.com/) with the unified package. For unified comparison, the referenced packages for raw materials and reagents were 100 g and 25 L, respectively.

b. Based on average exchange rates as of June 2022, 1 CNY was equal to 0.149 USD.

c. TCPG represent the total costs per gram of adsorbent.

* represent the supposed productivity (80%) of the samples.

References

(1) Sun, X.; Miao, J.; Xiao, J.; Xia, Q.; Zhao, Z. Heterogeneity of Adsorption Sites and Adsorption Kinetics of n-Hexane on Metal−Organic Framework MIL-101(Cr). *Chinese J. Chem. Eng.* **2014**, 22, 962-967.

(2) Hu, P.; Hu, J.; Wang, H.; Liu, H.; Zhou, J.; Liu, Y.; Wang, Y.; Ji, H. One-Step Ethylene Purification by an Ethane-Screening Metal-Organic Framework. *ACS Appl Mater Interfaces* **2022**, 14, 15195-15204.

(3) Hu, P.; Wang, H.; Xiong, C.; Liu, H.; Han, J.; Zhou, J.; Zhao, Z.; Wang, Y.; Ji, H. Probing the Node Chemistry of a Metal-Organic Framework to Achieve Ultrahigh Hydrophobicity and Highly Efficient CO_2/CH_4 Separation. *ACS Sustain. Chem. Eng.* **2021**, 9, 15897-15907.

(4) Krishna, R. The Maxwell−Stefan description of mixture diffusion in nanoporous crystalline materials. *Micropor. Mesopor. Mat.* **2014**, 185, 30-50.

(5) Krishna, R. Methodologies for evaluation of metal−organic frameworks in separation applications. *RSC Adv*. 2015, **5**, 52269-52295.

(6) Krishna, R. Screening metal−organic frameworks for mixture separations in fixed-bed adsorbers using a combined selectivity/capacity metric. *RSC Adv.* **2017**, 7, 35724-35737.

(7) Krishna, R. Methodologies for screening and selection of crystalline microporous materials in mixture separations. *Sep. Purif. Technol.* **2018**, 194, 281-300.

(8) Krishna, R. Metrics for Evaluation and Screening of Metal−Organic Frameworks for Applications in Mixture Separations. *ACS Omega* **2020**, 5, 16987-17004.

(9) Hu, P.; Han, J.; Zhou, J.; Wang, H.; Xiong, C.; Liu, H.; Zhou, X.; Wang, Y.; Ji, H. Customized H-bonding acceptor and aperture chemistry within a metal-organic framework for efficient C_3H_6/C_3H_8 separation. *Chem. Eng. J.* **2021**, 426, 131302.

(10) Wang, Y.; Huang, N. Y.; Zhang, X. W.; He, H.; Huang, R. K.; Ye, Z. M.; Li, Y.; Zhou, D. D.; Liao, P. Q.; Chen, X. M.et al. Selective Aerobic Oxidation of a Metal−Organic Framework Boosts Thermodynamic and Kinetic Propylene/Propane Selectivity. *Angewandte Chemie International Edition* **2019**, 58, 7692-7696.

(11) Wang, X.; Zhang, P.; Zhang, Z.; Yang, L.; Ding, Q.; Cui, X.; Wang, J.; Xing, H. Efficient Separation of Propene and Propane Using Anion-Pillared Metal−Organic Frameworks. *Ind. Eng. Chem. Res.* **2020**, 59, 3531-3537.

(12) Chen, Y.; Wu, H.; Lv, D.; Yuan, N.; Xia, Q.; Li, Z. A pillar-layer metal-organic framework for efficient adsorption separation of propylene over propane. *Sep. Purif. Technol.* **2018**, 204, 75-80.

(13) Chen, Y.; Qiao, Z.; Lv, D.; Duan, C.; Sun, X.; Wu, H.; Shi, R.; Xia, Q.; Li, Z. Efficient adsorptive separation of C3H6 over C3H8 on flexible and thermoresponsive CPL-1. *Chem. Eng. J.* **2017**, 328, 360-367.

(14) Yoon, J. W.; Kim, A.; Kim, M. J.; Yoon, T.; Kim, J.; Bae, Y. Low-temperature Cu(I) loading on a mesoporous Metal−Organic framework for adsorptive separation of C_3H_6/C_3H_8 mixtures. *Micropor. Mesopor. Mat.* **2019**, 279, 271-277.

(15) Zhang, Z.; Ding, Q.; Cui, X.; Jiang, X.; Xing, H. Fine-Tuning and Selective-Binding within an Anion-Functionalized Ultramicroporous Metal−Organic Framework for Efficient Olefin/Paraffin Separation. *ACS Appl. Mater. Inter.* **2020**, 12, 40229-40235.

(16) Wang, X.; Zhang, P.; Zhang, Z.; Yang, L.; Ding, Q.; Cui, X.; Wang, J.; Xing, H. Efficient Separation of Propene and Propane Using Anion-Pillared Metal−Organic Frameworks. *Ind. Eng. Chem. Res.* **2020**, 59, 3531-3537.

(17) Gao, J.; Cai, Y.; Qian, X.; Liu, P.; Wu, H.; Zhou, W.; Liu, D. X.; Li, L.; Lin, R. B.; Chen, B. A Microporous Hydrogen−Bonded Organic Framework for the Efficient Capture and Purification of Propylene. *Angewandte Chemie International Edition* **2021**, 60, 20400-20406.

(18) Zeng, H.; Xie, M.; Wang, T.; Wei, R.; Xie, X.; Zhao, Y.; Lu, W.; Li, D. Orthogonal-array dynamic molecular sieving of propylene/propane mixtures. *Nature* **2021**, 595, 542-548.

(19) Wang, Y.; Huang, N.; Zhang, X.; He, H.; Huang, R.; Ye, Z.; Li, Y.; Zhou, D.; Liao, P.; Chen, X.et al. Selective Aerobic Oxidation of a Metal-Organic Framework Boosts Thermodynamic and Kinetic Propylene/Propane Selectivity. *Angew. Chem. Int. Edit.* **2019**, 58, 7692-7696.

(20) Chen, Y.; Wu, H.; Lv, D.; Yuan, N.; Xia, Q.; Li, Z. A pillar-layer metal-organic framework for efficient adsorption separation of propylene over propane. *Sep. Purif. Technol.* **2018**, 204, 75-80.

(21) Chen, Y.; Wu, H.; Yu, L.; Tu, S.; Wu, Y.; Li, Z.; Xia, Q.Exploiting thermodynamic-kinetic synergetic effect for C_3H_6/C_3H_8 separation in pillar-layer MOFs. *Authorea.* **2021**.

(22) Lv, D.; Xu, J.; Zhou, P.; Tu, S.; Xu, F.; Yan, J.; Xi, H.; Liu, Z.; Yuan, W.; Fu, Q.et al. Highly selective separation of propylene/propane mixture on cost-effectively four-carbon linkers based metal-organic frameworks. *Chinese J. Chem. Eng.* **2022**.

(23) Tian, X.; Zhou, H.; Zhang, X.; Wang, C.; Qiu, Z.; Zhou, D.; Zhang, J. Two Isostructural Flexible Porous Coordination Polymers Showing Contrasting Single-Component and Mixture Adsorption Properties for Propylene/Propane. *Inorg. Chem.* **2020**, 59, 6047-6052.

(24) Geier, S. J.; Mason, J. A.; Bloch, E. D.; Queen, W. L.; Hudson, M. R.; Brown, C. M.; Long, J. R. Selective adsorption of ethylene over ethane and propylene over propane in the metal − organic frameworks M_2(dobdc) (M = Mg, Mn, Fe, Co, Ni, Zn). *Chem. Sci.* **2013**, 4, 2054.
